# Structural disorder of plasmid-encoded proteins in Bacteria and Archaea

**DOI:** 10.1186/s12859-018-2158-6

**Published:** 2018-04-25

**Authors:** Nenad S. Mitić, Saša N. Malkov, Jovana J. Kovačević, Gordana M. Pavlović-Lažetić, Miloš V. Beljanski

**Affiliations:** 1 Department of Computer Science, Faculty of Mathematics, University of Belgrade, P.O.B. 550 Studentski trg 16, Belgrade, 11001 Serbia; 2 Bio-lab, Institute of General and Physical Chemistry, P.O.B. 45, Studentski trg 12/V, Belgrade, 11001 Serbia

**Keywords:** Intrinsically disordered proteins, Plasmid-encoded proteins, Toxin/antitoxin, Bacteria and Archaea

## Abstract

**Background:**

In the last decade and a half it has been firmly established that a large number of proteins do not adopt a well-defined (ordered) structure under physiological conditions. Such intrinsically disordered proteins (IDPs) and intrinsically disordered (protein) regions (IDRs) are involved in essential cell processes through two basic mechanisms: the entropic chain mechanism which is responsible for rapid fluctuations among many alternative conformations, and molecular recognition via short recognition elements that bind to other molecules. IDPs possess a high adaptive potential and there is special interest in investigating their involvement in organism evolution.

**Results:**

We analyzed 2554 Bacterial and 139 Archaeal proteomes, with a total of 8,455,194 proteins for disorder content and its implications for adaptation of organisms, using three disorder predictors and three measures. Along with other findings, we revealed that for all three predictors and all three measures (1) Bacteria exhibit significantly more disorder than Archaea; (2) plasmid-encoded proteins contain considerably more IDRs than proteins encoded on chromosomes (or whole genomes) in both prokaryote superkingdoms; (3) plasmid proteins are significantly more disordered than chromosomal proteins only in the group of proteins with no COG category assigned; (4) antitoxin proteins in comparison to other proteins, are the most disordered (almost double) in both Bacterial and Archaeal proteomes; (5) plasmidal proteins are more disordered than chromosomal proteins in Bacterial antitoxins and toxin-unclassified proteins, but have almost the same disorder content in toxin proteins.

**Conclusion:**

Our results suggest that while disorder content depends on genome and proteome characteristics, it is more influenced by functional engagements than by gene location (on chromosome or plasmid).

**Electronic supplementary material:**

The online version of this article (10.1186/s12859-018-2158-6) contains supplementary material, which is available to authorized users.

## Background

Prokaryotic plasmids are extrachromosomal non-obligatory DNA molecules that replicate independently. They are transmitted between organisms by horizontal gene transfer and may be considered as mobile genetic elements, like transposons or prophages [1].

Plasmid backbone genes encode for proteins that are mostly involved in replication, copy number, partitioning, stability, etc. [2]. However, most plasmid genes encode for proteins with an unknown function. According to the Clusters of Orthologous Groups (COGs) classification, more than 25% of plasmid proteins have not been assigned to COGs [3]. Also, it was estimated that 13% of plasmid proteins belong to the so-called singleton ORFan category, consisting of proteins with no sequence homologies in other genomes, which are characterized by relatively short lengths, rapid evolution and are encoded by gene lower GC contents (it was shown that genes with a lower GC content tend to evolve at a faster rate as compared to genes with a higher GC content, although many other factors may also contribute to the evolutionary rate of proteins [2, 4]). These proteins have novel functions and are mostly annotated as hypothetical proteins of unknown function [5].

Aside from backbone genes, plasmids also contain genes that are involved in adaptive traits, such as the ability to exploit new environments or compounds, pathogenesis and antibiotic resistance. Of special interest are toxin/antitoxin genes and their products, because they often contribute to the maintenance of plasmids or genomic islands [6]. Toxin/antitoxin systems are found in plasmids and phages, as well as in chromosomes. They invade Bacterial genomes through horizontal gene transfer and participate in a wide range of cellular events, such as plasmid maintenance (via the mechanism of post-segregation killing), dormancy and persistence, phage defense, general stress response, etc. At present, toxin/antitoxin systems are classified according to their genetic structure and regulation into six types (I-VI) [7, 8]. They are composed of closely linked genes encoding a stable toxin, typically a low molecular weight protein, which causes growth arrest by inhibition of essential cellular processes (including DNA replication, translation, cell division, etc.), and its cognate labile antitoxin, which can either be a non-coding RNA (types I and III) or a small protein (types II, IV, V, and VI), which protects the host from the toxin’s deleterious effect. During normal growth conditions, the antitoxins must be constantly synthesized in order to inhibit their cognate toxins. The function of chromosomally encoded toxin/antitoxin systems is less clear [9]. In terms of their structure-function relationship, it is of special interest that antitoxins often lack a well-defined 3D structure, i.e. they are intrinsically disordered [7].

Intrinsically disordered proteins (IDPs) and intrinsically disordered (protein) regions (IDRs) within structured proteins are defined by the absence of a stable tertiary structure and a corresponding high degree of flexibility under physiological conditions [10]. IDPs usually lack rigid three-dimensional structures “due to diminished hydrophobic interactions determined by the specific amino acid (AA) compositions which are typically depleted in hydrophobic, order-promoting residues, but are enriched in polar and charged disorder-promoting residues” [11]. IDPs were recently reviewed in a special edition of Chemical Reviews [12] and described in detail in the monograph [13]. Since IDPs are a challenge to study experimentally, a number of prediction tools (currently, over 60) have been developed [14, 15].

IDPs perform their function via two basic mechanisms: (1) the entropic chain mechanism which is responsible for rapid fluctuations among many alternative conformations, providing different biological functions to IDPs (such as linkers, spacers, bristles or springs), and (2) by molecular recognition via short recognition elements, that bind to other molecules such as: performed structural elements, molecular recognition features, or short linear motifs [16]. Functional classification of proteins according to COGs shows that proteins belonging to the Metabolism group (Me) have a lower disorder content than proteins in Cellular processes and signaling (Cp) and Information storage and processing (Isp) groups [17], i.e. the structural disorder is enriched in proteins involved in signaling and regulatory functions and depleted in enzymes [18].

Taxonomically, IDPs are present in the proteomes of all of the three superkingdoms (Archaea, Bacteria and Eukarya), as well as in their viruses. The analysis of disorder content revealed that Bacteria have a slightly higher level of protein disorder than Archaea. Depending on the predictor and measure used, the disorder content varies in the range of 12 to 32% for Archaea, and 18 to 35% for Bacteria [17, 19, 20]. Eukarya generally contain higher disorder content, ranging from 35 to 50%, while in viruses the disorder content varies to a large extent from 2.9 to 23.1% [21].

The aim of this work was to examine protein disorder contents: (1) in Bacterial and Archaeal plasmids and to compare them with those in chromosomes; (2) in Bacterial and Archaeal plasmids and chromosomes as a function of genome size, proteome size, average protein length and GC percentage; (3) in plasmid-encoded proteins classified according to COGs, and (4) in toxin and antitoxin plasmid- and chromosome-encoded proteins, as a specific group of proteins with known functions. Our results suggest that while disorder content depends on genome and proteome characteristics, it is more influenced by functional engagements than by gene location (on chromosome or plasmid).

### Dataset

The dataset was collected in May 2015 from the NCBI database (currently available at ftp://ftp.ncbi.nlm.nih.gov/genomes/archive/old_refseq/Bacteria/) and the toxin/antitoxin database (http://202.120.12.135/TADB2/). Material downloaded from NCBI site includes COG functional classification of proteins. Only proteins that were already included in the downloaded material were selected from toxin/antitoxin database. In addition, we calculated a number of genome and proteome characteristics from the downloaded sequences; these included genome size, number of chromosomes, number of plasmids, the percentage of GC nucleotides, proteome size and average protein length.

The dataset included 2554 Bacterial and 139 Archaeal organisms with 2842 chromosomes (2703 in Bacteria, 139 in Archaea) and 2063 plasmids (2040 in Bacteria, 23 in Archaea). The maximum number of plasmids in a Bacterial organism is 39, in an Archaeal organism is 2. The distribution of organisms related to the number of plasmids and chromosomes is shown in Table 1. There are 8,455,194 different proteins – 8,158,660 Bacterial (7,919,866 chromosomal and 238,794 plasmidal) and 296,534 Archaeal (295,083 chromosomal and 1451 plasmidal). The Additional file 1: Figure S1 presents the distribution of protein number and average length over subsets of the material in the dataset.

**Table 1:** Organisms in the dataset

				Without plasmids			With plasmids		
	#Organisms	#Phyla	#Classes	Total	1 chr	> 1 chr	Total	1 chr (1pls/> 1pls)	>1 chr (1pls/> 1pls)
Total	2693	41	80	1796	1717	79	897	844 (434/410)	53 (28/25)
Archaea	139	6	17	119	119	0	20	20 (17/ 3)	0
Bacteria	2554	35	63	1677	1598	79	877	824 (417/407)	53 (28/25)

There are 12 organisms with 10 or more plasmids, with one chromosome each, 8 of which from the phylum *Spirochaetes*, one from the phylum *Proteobacteria*, and three from the phylum *Firmicutes*. There are 115 Bacterial organisms with 2 chromosomes and 17 Bacterial organisms with 3 chromosomes. All Archaeal organisms have exactly 1 chromosome

Proteins are assigned to COG categories (20 in total), which are further grouped in the COG groups as proteins participating in Cellular Processes (Cp), Information Storage and Processing (Isp), Metabolism (Me), as Poorly characterized (Pc) proteins and as proteins Not in COGs (N.C.) [3]. Proteins labeled as “unknown” (COG determined but not cited in the downloaded material) were added to the N.C. group (7161 Bacterial and 50 Archaeal). The protein distribution according to COG groups and categories is presented in Additional file 1: Figures S2 and S3, respectively. The total number of proteins in COG groups are slightly higher than the number of different proteins because there are proteins that have been assigned to more than one COG group or category.

There are 11,564 toxin/antitoxin proteins included in the dataset. The distribution of toxin/antitoxin proteins over COG groups in the subsets (chromosomes and plasmids) is shown in the Table 2 and Additional file 1: Table S1.

**Table 2:** Distribution of toxin/antitoxin/toxin-unclassified proteins over COG groups and over chromosomes and plasmids

	Cellular processing			Information storage and processing			Metabolism			Poorly characterized			Not in COGs		
	Avg. prot. Len.	Avg. % of disord. AAs	Num. of proteins	Avg. prot. Len.	Avg. % of disord. AAs	Num. of proteins	Avg. prot. Len.	Avg. % of disord. AAs	Num. of proteins	Avg. prot. Len.	Avg. % of disord. AAs	Num. of proteins	Avg. prot. Len.	Avg. % of disord. AAs	Num. of proteins
Archaea															
chromosome															
toxin	86.48	26.543	47	99.32	22.849	46	242	6.198	1	130.14	11.476	328	116.06	15.344	126
antitoxin	77.8	27.634	10	88.57	36.007	47	629	0.476	1	113.67	17.169	243	88.37	37.091	218
unclassified	353.48	11.602	23,786	292.79	13.823	35,181	346.28	8.793	65,161	290.67	10.437	43,957	246.85	12.514	137,096
plasmid															
toxin	–	–	0	–	–	0	–	–	0	153	4.575	1	–	–	0
antitoxin	–	–	0	–	–	0	–	–	0	–	–	0	68	8.823	1
unclassified	482.52	17.609	100	449.56	21.676	198	347.38	20.904	55	401.25	18.528	98	234.35	21.621	1042
Bacteria															
chromosome															
toxin	109.2	21.259	566	134.46	19.733	217	210.61	13.186	114	144.9	15.596	3127	120.5	19.746	1021
antitoxin	87.32	42.618	481	115.7	36.092	1493	378.66	11.817	12	106.33	32.195	1336	107.75	37.682	1549
unclassified	388.79	17.384	815,045	329.47	17.496	695,033	377.01	11.161	1,435,900	303.64	15.268	738,635	289.74	16.839	4,584,662
plasmid															
toxin	109.36	23.104	41	110.57	23.417	19	256.61	13.998	13	136.46	17.357	243	139.3	17.328	86
antitoxin	86.45	48.819	48	99.33	43.268	99	–	–	0	111.05	39.203	106	111.2	41.965	130
unclassified	399.3	17.976	17,210	326.23	18.968	19,305	386.8	11.417	29,551	313.49	16.074	15,407	256.96	20.259	165,363

## Methods

### Intrinsically disordered proteins

We could not use data from databases containing pre-calculated disorder level (such as [22, 23]) because of the small intersection of protein sets in our material and in these databases. For example, MobiDB includes only 5% of proteins from our dataset (comparison was done by using corresponding UniProt ids). The disorder level for each residue of each protein in our dataset was calculated using three different disorder predictors: PONDR VSL2b® [24], IsUnstruct [25] and IUpred-L [26].

These predictors are widely used and are based on different approaches. VSL2b is a combination of neural network predictors for both short and long disordered regions. IsUnstruct is based on an approximation of the Ising model, a mathematical model of ferromagnetism in statistical mechanics, using penalty for changing between ordered/disordered states among neighboring amino acids; IUPred-L (long) assigns a disorder score to an amino acid based on the pairwise interaction energy score. Since the VSL2b predictor predicts well both short and long disordered regions while the IUPred-L predicts long disordered regions better than short ones, it is expected that the former will predict a higher disorder content than the latter (as is the case in the D2P2 database (http://d2p2.pro/)). The disorder content predicted by IsUnstruct is between these two. Predictions were performed for all 8,455,194 proteins using IUPred-L and IsUnstruct predictors, whereas VSL2b performed predictions for 8,448,127 proteins (since other protein sequences contain some amino acid tags that VSL2b does not recognize). Haloarchaean proteomes, due to adaptive pressure, have specific AA contents, which lead to IDP prediction errors as revealed by Xue et al. [19, 20], and Syutkin and all [27], and were accordingly excluded from the analysis.

We calculated three measures of protein disorder content in Bacteria and Archaea proteomes in three data collections: complete genomes, chromosomes and plasmids. The first measure is the averaged fraction of disordered AAs by proteins in a proteome (percentage of all predicted disordered AAs in a protein and then averaged by all the proteins in the proteome). The second measure is the percentage of AAs in long (> 30 AA) disordered regions; this was averaged over all of the proteins in a proteome. The last measure is the percentage of proteins (in a proteome) with at least one long disordered region. Having calculated the disorder of a proteome, disorder of a collection of proteomes (set of organisms, set of chromosomes, set of plasmids) was calculated as the average disorder over all the proteomes in the collection [28, 29].

### Disorder content of different COG groups

Functional classification by COGs is the result of protein sequence homology, implying their structural and thus functional similarity. We chose the COG functional classification (among different existing ones) because most genomes are COGged and COG annotations are easily accessible [3]. We extended our previous research on COG-related disorder to three separate data subsets - complete genomes, chromosomes and plasmids from the superkingdoms Bacteria and Archaea, and COG functional groups and categories (A-Z). The main reason for this type of analysis was to determine the sources of (possible) different levels of disorder in proteomes of different DNA molecules (chromosomes, plasmids) and complete genomes, i.e. whether there is an increased (or decreased) number of proteins in disorder-abundant COGs, or disorder-abundant (or depleted) content of proteomes in general.

Since a large number of proteins belong to the “Not in COG” (N.C.) group, we repeated the complete analyses for a reduced dataset that consisted of “mostly COGged” organisms so as to be able to compare and verify the results obtained for the whole dataset. We analyzed only those organisms where the total length of proteins in the N.C. group was at most 20% of their total proteome length. The selected subset includes 4,332,156 proteins. Number of organisms, chromosomes and plasmids in the subset is shown in Additional file 1: Table S2.

### Statistical analysis

All the calculations (average protein length, GC percent, etc.) were performed on a per- organism bases. The same also holds for plasmids and chromosomes. In order to investigate the linear (or at least monotonic) relationship between different phenomena, we calculated Pearson’s linear correlation coefficients. The difference in the distribution of the disorder content among different data collections was tested using the Mann-Whitney-Wilcoxon U test of equality of medians and Student’s t-test of equality of means. The impact that that different attributes have on protein disorder is estimated by developing a disorder prediction model using IBM InfoSphere Warehouse Intelligent Miner. Intelligent Miner is IBM’s commercial data mining software included in InfoSphere® Warehouse which is a suite of products that combines the strength of DB2 with a data warehousing infrastructure from IBM® (https://www.ibm.com/). It includes variety of algorithms for mining association rules, clustering, classification (prediction), sequential patterns, regression, and time series. IBM Intelligent Miner can perform mining functions against traditional relational databases or flat files, and is able to work with large quantity of data that cannot fit into memory. Prediction algorithm generates, as a component of prediction model, an estimation of the impact of the input components on model, which is in this research used to estimate impact of protein characteristics on protein disorder.

## Results and Discussion

### Disorder content of Bacteria and Archaea

**Fig. 1:**
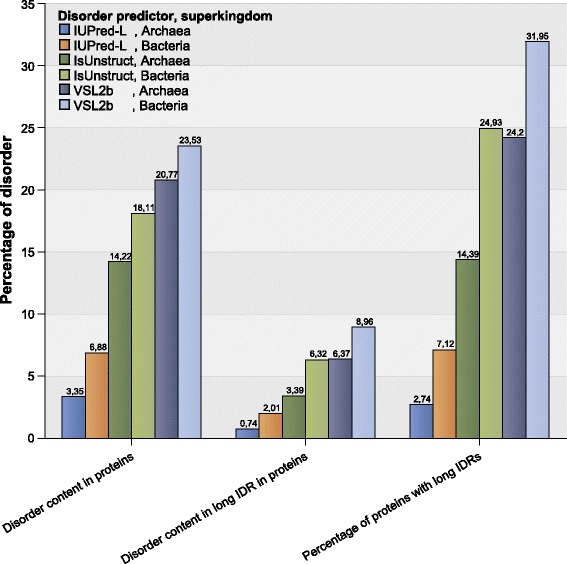
Disorder content in Archaea and Bacteria. Disorder content is predicted using three predictors (IUPred-L, IsUnstruct and VSL2b) and three measures

The results of disorder content analysis in Bacteria and Archaea were generally in accordance with our previous findings [17] and the results of others (e.g. [22]). For all three predictors and all three measures, Bacteria exhibit significantly more disorder than Archaea (ranging on average from 6.88 to 23.53% for Bacteria and 3.35 to 20.77% for Archaea, for the percentage of disordered AAs and different predictors; similar results were obtained for other measures, see Fig. 1). The Student’s t-test for equality of means resulted in a *p*-value < 0.01. The absolute values differed among the predictors and among the measures, but the relationship between the disorder content in Bacteria and Archaea generally remained the same.

**Fig. 2:**
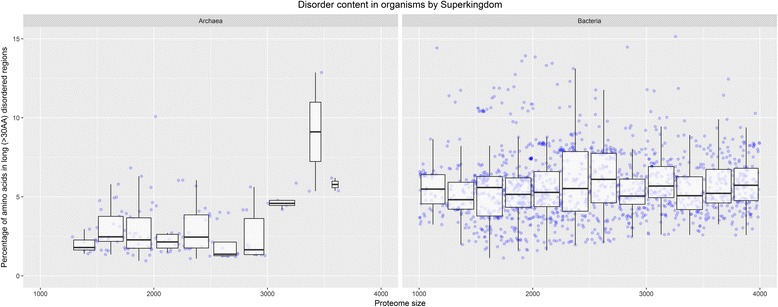
Disorder content in long (>30AA) disordered regions in Bacteria and Archaea with small proteomes. The disorder content represents the percentage of amino acids in long disordered regions, predicted by the IsUnstruct predictor. Since Archaea proteome size is in range of 1000 to 4000 proteins, only Bacteria in the same range are selected, in order to emphasize the difference in predicted disorder content between Bacteria and Archaea with similar proteome sizes. The box diagrams in the paper follow the usual representation: 1) the horizontal line inside a box represents the median value (50% of the samples is lower and 50% of the samples are higher than median); 2) lower box bound represents *first quartile* value (25% of data are lower and 75% are higher than first quartile); 3) upper box bound represents *third quartile* value (75% of data are lower and 25% are higher than third quartile); 4) the box height represents *interquartile range* (IQR); in the case of normal distribution, IQR = 1.35 x σ; 5) the whiskers (vertical lines above and under the box) ranges up to the highest datum within 1.5 x IQR of the upper quartile and down to the lowest datum within 1.5× IQR of the lower quartile; 6) the dots above the top whisker and under the bottom whisker represent outliers, i.e. the samples that are out of the range (in some of the diagrams each sample is represented as a dot, and outliers are not specifically highlighted, because it is obvious which samples lay out of the whiskers range); 7) in some of the diagrams the red dot represents the mean value

This relationship was confirmed by the high values obtained for Pearson’s correlation coefficients for different measures of disorder and different disorder predictors (correlation coefficients ranging from 0.88 to 0.98 for different measures on the same predictors and from 0.74 to 0.81 for different predictors and the same measure). The difference in disorder content in Bacteria and Archaea is not a consequence of different proteome sizes – we compared Archaea with subset of Bacterial proteomes with similar proteome sizes (up to 4000 proteins) and observed the same difference in disorder content in favor of Bacteria (see Fig. 2).

In further analysis we applied all three predictors and used all three (highly correlated) disorder measures; however, for clarity, we have presented in the main text each result by just one predictor and one measure (we used the percentage of AAs in long (> 30 AA) disordered regions, unless otherwise specified), while some results for other predictors and measures are presented in Additional file.

### Disorder content of chromosomes and plasmids

**Fig. 3:**
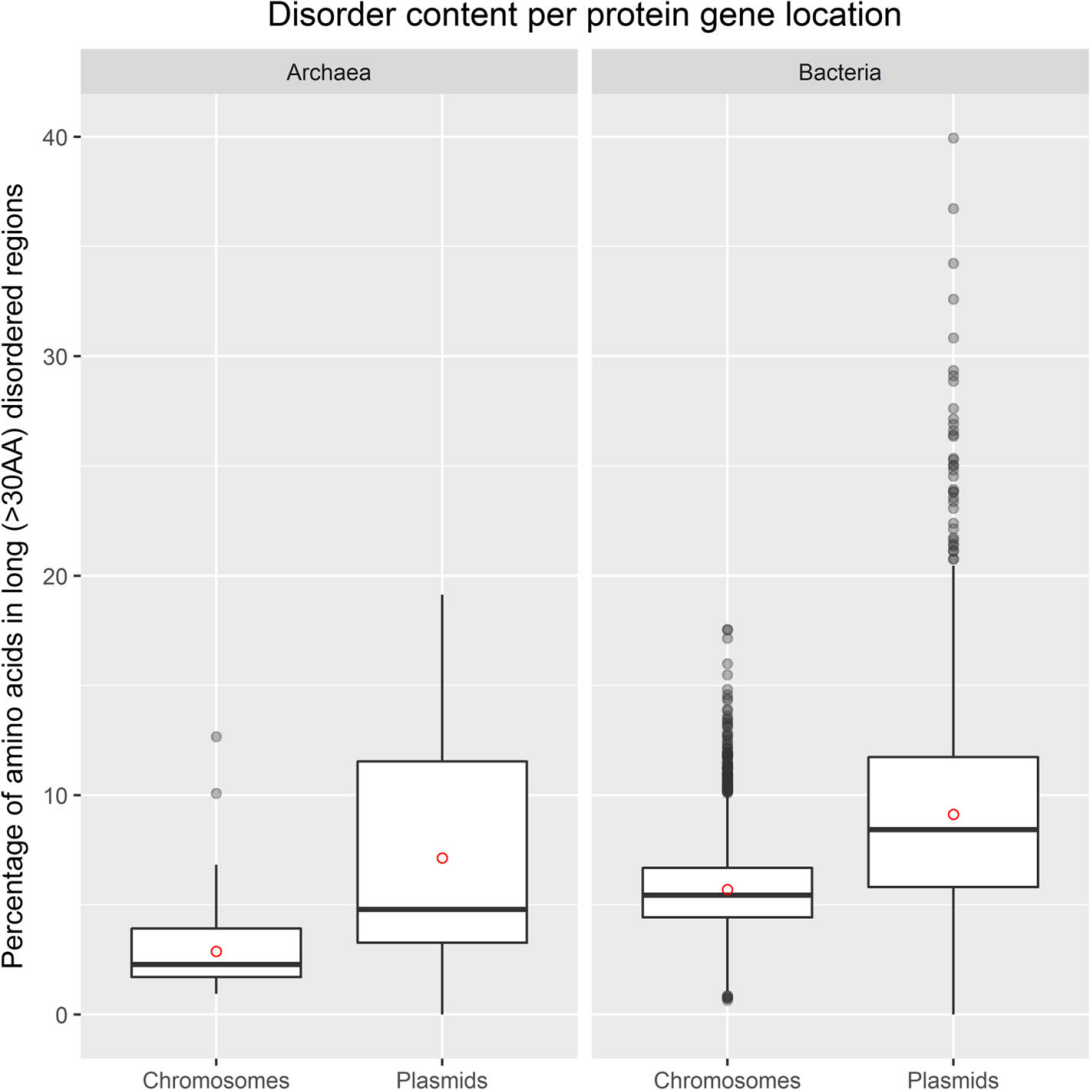
Disorder content in long (>30AA) disordered regions in Bacteria and Archaea per gene location. The disorder content represents the percentage of amino acids in long disordered regions, predicted by the IsUnstruct predictor. The proteomes are divided in protein sets encoded by chromosome/plasmid DNA. The overall organisms disorder content is almost the same as in the chorosome-encoded proteome subset

A comparative analysis of the disorder content in proteins encoded by plasmids and chromosomes was performed for the first time. It revealed that in both Bacteria and Archaea plasmid-encoded proteins contain considerably more IDRs than proteins encoded on chromosomes (Fig. 3 represents these findings for long disorder measure and the IsUnstruct predictor; similar findings for all the three measures and all the three predictors, for different data subsets - plasmids, chromosomes, genomes with and without plasmids, are presented in Additional file 1: Figure S4). These findings are statistically significant according to the Mann-Whitney nonparametric test and Student’s t-test (for the IsUnstruct predictor and the percentage of disordered AAs, the *p*-value < 0.00001). Also, the range of IDR content is much larger for plasmid encoded proteins in comparison to chromosome encoded ones (0 to 40 and 2 to 17% for plasmids and chromosomes, respectively).

Relatively wide range of IDP content was also observed for viral and bacteriophagal proteomes [20]. Many of them have high IDP content, especially those with increased proteome size, which is similar to plasmids [20, 30]. In order to enable replication, viral proteomes have been shaped by interactions with the host proteome, i.e. they have evolved to mimic host cellular processes and to interfere with them. This is possible due to the higher content of IDPs [20] because of their special functional attributes, as observed in viral proteins which display a high occurrence of disordered segments, a feature that might endow viral proteins with increased structural flexibility and effective ways to interact with host components [31]. The increased disorder content in plasmids is thus not surprising since both plasmids and phages need to be incorporated into a living cell and utilize host molecular machine in order to proliferate [32].

### Disorder content of chromosomes and plasmids vs. genome and proteome characteristics

**Fig. 4:**
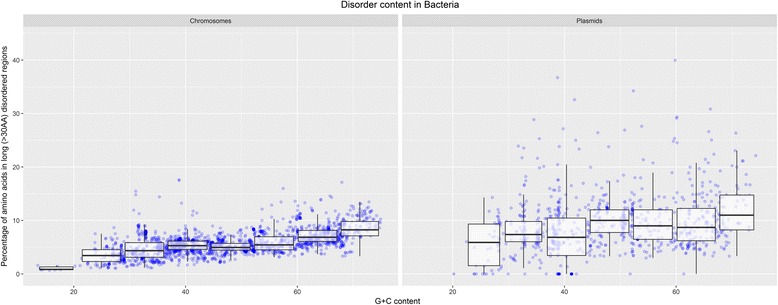
Disorder content in long (>30AA) disordered regions in Bacteria by gene location, as a function of G + C content. Disorder is predicted by the IsUnstruct predictor

Our detailed analysis of proteins encoded by Bacterial chromosomes and plasmids revealed a general increase in disorder content as a function of genome size, G + C content and proteome size, while average protein length exhibits less obvious relationship to disorder level (Fig. 4 represents these findings for G + C content, long disorder measure and the IsUnstruct predictor; results for other three characteristics - genome size, proteome size and average protein length, for the same disorder measure and the IsUnstruct predictor, for both Archaea and Bacteria, are presented in Additional file 1: Figure S5). Similar holds for Archaeal chromosomes and plasmids, although this trend is less expressed, due to smaller number of Archaeal genomes, as well as smaller range of the corresponding characteristics (proteome size, G + C content and especially genome size).

Specifically, there is an apparent increase in disorder content for G + C content larger than 50%, that can be explained by the fact that a high percentage of GC in codons results in an increased presence of disorder promoting amino acids (such as Gly, Ala, Arg, and Pro) [17, 18]. The relatively uniform disorder content for genomes that have a G + C content between 30 and 50% can be explained by the selective alteration in the G + C content on third and first positions in codons, and consequently only a change in codon usage and not in AA usage. As it concerns proteome size, a larger proteome implies more complex interaction networks and thus higher disorder content, since one of the main functions of IDPs is in molecular interaction and recognition.

Correlation analysis shows a statistically significant positive linear correlation between disorder content of Bacterial chromosome and plasmid proteomes and each of the genome/proteome characteristics - G + C content, proteome and genome size and average protein length, except for average protein length of plasmids. Archaeal chromosomal proteomes exhibit statistically significant correlation between disorder content and G + C content, genome and proteome size. Archaeal plasmids (the sample being rather small) do not exhibit any significant correlations with genome/proteome characteristics except for G + C content (see Table 3).

**Table 3:** Statistical correlation between predicted disorder content and organism characteristics

			Bacteria					Archaea				
			Complete	Seg1	Seg2	Seg3	Seg4	Complete	Seg1	Seg2	Seg3	Seg4
Chromosomes	Avg. protein len.	Correlation coef.	**0.1042**	−0.1278	**−0.0714**	**0.1220**	**0.2643**	0.1480	−0.3819	**0.3125**	0.1829	–
		Sample size	2554	40	921	1504	89	139	6	124	9	0
		Significance of CC	< 0.0001	0.4319	0.0303	< 0.0001	0.0123	0.0821	0.4550	0.0004	0.6376	–
	G + C content	Correlation coef.	**0.6060**	**0.3054**	**0.2793**	**0.2741**	**0.3052**	**0.2667**	−1.000	0.0653	0.1818	0.7369
		Sample size	2554	151	1043	756	604	139	2	77	54	6
		Significance of CC	< 0.0001	0.0001	< 0.0001	< 0.0001	< 0.0001	0.0015	–	0.5726	0.1883	0.0947
	Proteome size	Correlation coef.	**0.2950**	**0.1345**	**0.0689**	**0.3442**	0.2377	**0.2978**	0.0817	**0.5330**	–	–
		Sample size	2554	1128	1118	276	32	139	115	24	0	0
		Significance of CC	< 0.0001	< 0.0001	0.0212	< 0.0001	0.1902	0.0004	0.3854	0.0073	–	–
	Genome size	Correlation coef.	**0.3019**	**0.1592**	**0.1562**	0.1159	0.8357	**0.3585**	**0.3341**	−0.8534	–	–
		Sample size	2554	1469	995	87	3	139	136	3	0	0
		Significance of CC	< 0.0001	< 0.0001	< 0.0001	0.2851	–	< 0.0001	< 0.0001	–	–	–
Plasmids	Avg. rotein len.	Correlation coef.	−0.0570	0.7456	0.0207	**−0.1596**	0.2914	0.0408	/	−0.0671	−1.0000	/
		Sample size	877	4	371	491	11	20	1	17	2	0
		Significance of CC	0.0916	–	0.6911	0.0004	0.3846	0.8644	–	0.7980	–	–
	G + C content	Correlation coef.	**0.3324**	**0.4513**	0.0693	0.0844	**0.3494**	**0.5399**	0.5155	0.0494	−0.6586	/
		Sample size	877	123	319	230	205	20	6	8	5	1
		Significance of CC	< 0.0001	< 0.0001	0.2171	0.2022	< 0.0001	0.0140	0.2952	0.9075	–	–
	Proteome size	Correlation coef.	**0.1976**	**0.4958**	0.0008	**−0.1792**	**0.4609**	0.0863	0.0866	0.1977	/	/
		Sample size	877	215	392	238	32	20	13	7	0	0
		Significance of CC	< 0.0001	< 0.0001	0.9874	0.0056	0.0079	0.7175	0.7785	0.6709	–	–
	Genome size	Correlation coef.	**0.2048**	**0.4079**	0.0518	0.1335	**0.5414**	0.0645	−0.1670	−0.9999	/	/
		Sample size	877	259	460	137	21	20	17	3	0	0
		Significance of CC	< 0.0001	< 0.0001	0.2676	0.1199	0.0113	0.7870	0.5218	–	–	–

The table represents the statistical correlation between predicted disorder content and different organism characteristics. The disorder content is predicted using IsUnstruct predictor and measured as a percentage of amino acids in long disordered regions (> = 30AA)

For each sample set (Archaeal/Bacteral chromosomes, plasmids) and each of the observed characteristics, the samples are additionally classified in 4 segments (quarters) by range of the observed characteristics. Correlations are computed for the whole sample and additionally for each of the segments, to find out if the correlation is stronger for some segment (quarter) of the characteristics’ range. The significant correlations are emphasized in boldface

### Disorder content in different COG groups in chromosomes and plasmids

**Fig. 5:**
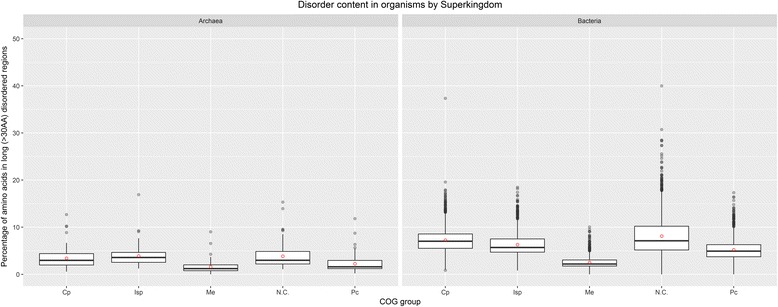
Disorder content in long (>30AA) disordered regions for different clusters of orthologous groups of proteins (COG groups) in Archaea and Bacteria. Disorder is predicted by the IsUnstruct predictor. COG groups are: Cp – Cellular Processes, Isp – Information Storage and Processing, Me –Metabolic, N.C. – Not in COG, Pc – Poorly characterized. The box diagrams in the paper follow the usual representation (see Fig. 2 caption for details)

Our analysis showed that in both Bacteria and Archaea complete proteomes the Metabolism (Me) COG group of proteins has the lowest disorder content among all COG groups, while Not in COGs (N.C.) and Poorly characterized (Pc) are abundant in IDR content. Figure 5 presents the overall long-disorder level per COG groups of proteins in Archaea and Bacteria, obtained by the IsUnstruct predictor. Additional file 1: Figure S6 represents the corresponding data for all the three measures.

**Fig. 6:**
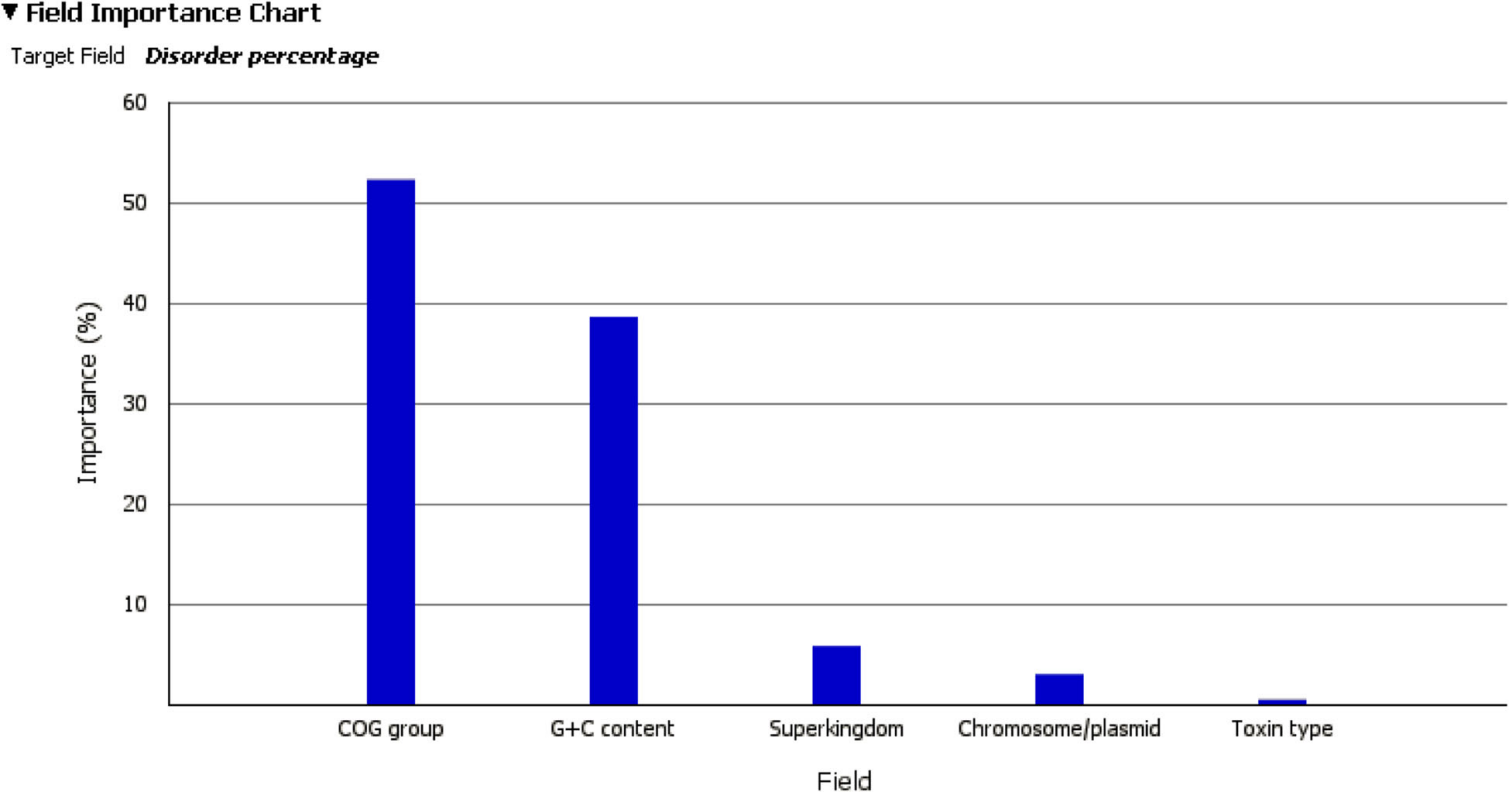
Impact of the attributes on disorder content, Variable COG denotes a COG group of a gene/protein (similarly for GC, Superkingdom. Toxin type, Chromosome/plasmid). Bar sizes denote level of impact of each characteristics on protein disorder. “Importance” on the diagram actually means impact. The highest impact on the percentage of protein disorder has COG group (N.C., Cp, Isp, Pc, Me) the protein belongs to (52.25%), then the percentage of GC nucleotides (38.60%), while impact of other characteristics is considerably lower (Superkingdom - 5.78%, Chromosome/plasmid - 2.96% i Toxin type - 0.41%)

Impact of different protein characteristics (super kingdom, chromosome/plasmid, COG group, toxin type) on protein disorder is represented through a data mining model for prediction percentage of protein disorder based on the specified organism characteristics. Prediction is obtained by using the IBM Intelligent Miner tool which identifies the characteristics having the highest impact on the prediction model. Figure 6 graphically represents impact of specific characteristics used in the model for predicting percentage of protein disorder. The results show that the COG classification has the highest impact on disorder content, even higher than G + C content.

If we consider the chromosome- and plasmid-encoded proteins separately with respect to COG groups, then the overall increased level of disorder in plasmid-encoded proteins could have two different causes:

- because plasmids are abundant in proteins in COG functional groups with higher disorder, or
- because the disorder level per protein is higher in plasmid proteins than in chromosome proteins in the same COG groups.

**Fig. 7:**
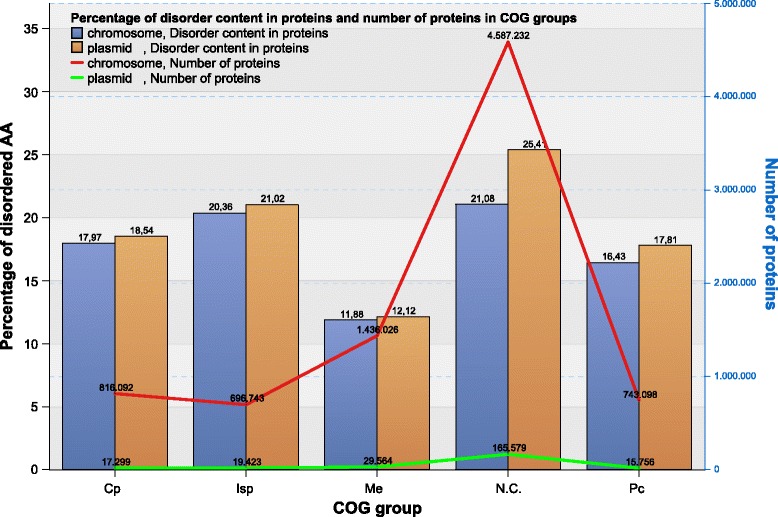
Disorder content of Bacterial COG groups in plasmids and chromosomes expressed as the percentage of disordered AAs

The obtained results show that:

- Plasmids are not abundant in proteins classified in COG groups with higher disorder, except for the Not in COGs (N.C.) group (69% in plasmidal vs 56% in chromosomal proteins), as shown in Fig. 7. Additional file 1: Figure S7 presents the distribution of proteins per COG groups in more detail.
- Plasmid proteins are more disordered than chromosomal proteins in the N.C. group, as also shown in Fig. 7 for the Is Unstruct predictor and percentage of disordered AA (the corresponding results for other predictors and measures are presented in Additional file 1: Figure S8). The result is statistically significant (Student’s t-test, *p* value < 0.05).

**Fig. 8:**
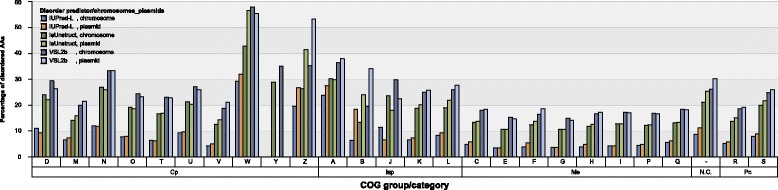
Disorder content of different COG categories and data subsets for Bacteria. Plasmid-encoded proteins in Not in COG (N.C.) and Poorly characterized (Pc) groups have higher disorder content than chromosome-encoded ones, while in most of the categories in Cellular processes (Cp), Information storage and processing (Isp) and Metabolism (Me) COG groups, plasmid-encoded proteins have similar or lower disorder content than chromosome-encoded ones (Cell motility (N), Cell cycle control, cell division, chromosome partitioning (D) and Intracellular trafficking, secretion, and vesicular transport (U) COG categories in Cp group, Translation, ribosomal structure and biogenesis (J) COG category in Isp group, Energy production and conversion COG (C), Amino acid transport and metabolism (E), Carbohydrate transport and metabolism (G), Lipid transport and metabolism (I), Inorganic ion transport and metabolism (P) and Secondary metabolites biosynthesis, transport, and catabolism (Q) in Me group. For all measures and Archaea see Additional file 1: Figure S9

Plasmids encode for a small number of proteins in all the COG groups and categories, except in N.C. group. IDR content in plasmid encoded proteins is higher or similar as in chromosome encoded proteins for all COG categories (see Fig. 8 for percentage of disordered AA per COG categories in Bacteria; similar data for other measures and for Archaea are presented in Additional file 1: Figure S9). Disorder content of Bacterial and Archaeal COG groups and categories reveals similar distribution, however, due to significantly smaller protein sample of Archaea they will not be discussed further, except for the N.C. group of proteins. According to ACLAME database [2] on plasmid encoded proteins, main functional categories found on plasmids belong to Isp and Cp COG groups, almost twice as many proteins as in functional categories in Me COG group. This may suggest the functions of N.C. group proteins in our dataset.

**Fig. 9:**
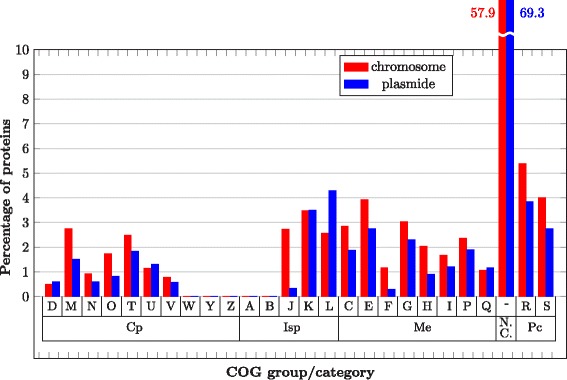
Percentage of proteins in COG categories for Bacteria For exact data and the distribution of proteins in COG categories for Archaea, see Additional file 1: Figure S3

**Fig. 10:**
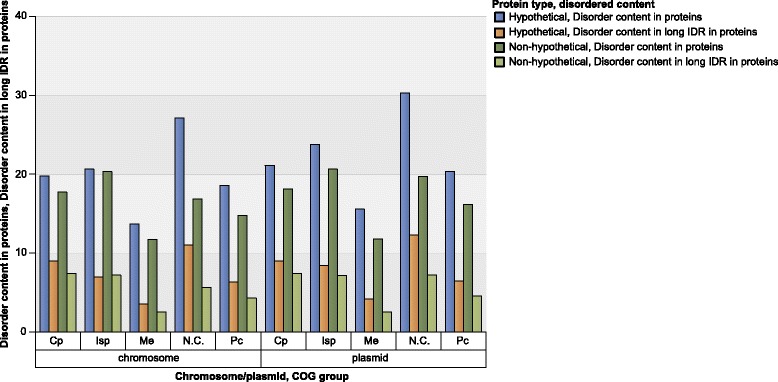
Disorder content in hypothetical proteins in comparison to non-hypothetical proteins for Bacteria. For Archaea and exact data, Additional file 1: Figure S10

Further analysis of proteins not categorized according to COGs (N.C. group) in chromosomes and plasmids revealed that:

1. In Bacteria and Archaea, proteins belonging to N.C. group are most abundant among both chromosome and plasmid encoded proteins, as presented in protein distribution according to COG groups and categories for Bacteria in Fig. 9 (see Additional file 1: Figure S3 for Archaea and detailed data).
2. The average length of proteins in the N.C. group is lower in comparison to other COG groups, for both chromosome encoded and plasmid encoded proteins. The majority of N.C. proteins from Bacterial plasmids and both Archaeal plasmids and chromosomes, are hypothetical. The fraction of hypothetical proteins encoded by Bacterial chromosomes in the N.C. group is lower than the fraction of non-hypothetical proteins (41 and 59%, respectively). The opposite holds for Bacterial plasmids (54 and 46% respectively). The most of all hypothetical proteins belong to N.C. group, i.e. 77% for Bacterial chromosomes encoded proteins (Table 4).
3. In N.C. group, the average length of hypothetical proteins is much smaller in comparison with non-hypothetical proteins (i.e. for Bacterial chromosome encoded proteins the ratio is 210/345 AA, and for Bacterial plasmid encoded proteins the ratio is 192/334 AA). The differences are not so distinct for proteins in other COG groups (Table 4).
4. Bacterial hypothetical proteins in the N.C. group contain 61 - 96% higher disorder contents than non-hypothetical proteins, depending on the disorder measure (see Fig. 10 for Bacteria, and Additional file 1: Figure S10 for Archaea and detailed data).

**Table 4:** Percentage of hypothetical proteins categorized and not categorized in COGs

		Not categorized in COGs						Categorized in COGs					
		# of proteins	# of hypothetical proteins	% of hypothetical proteins	Avg. protein length	Avg. protein length of hypothetical proteins	Avg. protein length of non-hypothetical proteins	# of proteins	# of hypothetical proteins	% of hypothetical proteins	Avg. protein length	Avg. protein length of hypothetical proteins	Avg. protein length of non-hypothetical proteins
Archaea	chromosome	137,440	80,722	58.73%	246.48	199.36	313.54	168,808	36,904	21.8%	320.69	272.03	334.30
	plasmid	1043	882	84.56%	234.19	203.22	403.84	452	114	25.22%	433.29	427.13	435.36
Bacteria	chromosome	4,587,232	1,889,430	41.19%	289.64	210.22	345.27	3,691,959	564,229	15.28%	355.48	299.67	365.55
	plasmid	165,579	89,488	54.04%	256.78	191.62	333.41	82,042	13,326	16.24%	359.56	312.21	368.74

Note: Total number of proteins is greater than number of proteins in material (8.455.194) because some proteins belongs to more than one COG category

It was estimated that 20–30% of Bacterial genomes are comprised of ORFan genes. Most of these genes are expressed, they have lower GC contents, differ in codon usage and have evolved faster. ORFan proteins are relatively small, with a specific AA composition, etc. At the functional level, ORFan proteins are associated with regulatory, growth- and transport-related processes [33–35]. Mukherjee et al. [36] found that ORFan genes encode unstructured proteins with a significantly higher fraction of disordered AAs as compared to nonORFan genes.

These results are in agreement with our results related to the disorder content in plasmid-encoded proteins, their short lengths and high representation in the N.C. group, especially with the high presence of hypothetical proteins in the N.C. group. We may conclude that the abundance of IDRs in plasmids is influenced by the fact that the most of plasmid proteins belong to N.C. group, which is rich in IDR content and hypothesize that the proteins in N.C. and Pc groups of proteins, could represent products of fast evolving genes within organisms and/or could have been acquired by horizontal gene transfer (HGT) by plasmids or phages from still unknown Bacterial species. HGT plays an important role in Bacterial and Archaeal evolution (it is estimated that as much as 81% of genes have been acquired by HGT) [37, 38]. Plasmids undergo fast rate of sequence turnover and represent key vectors of genetic exchange between Bacterial genomes [39]. This may explain a high number of N.C. proteins in both Bacterial and Arhaeal plasmids and chromosomes.

Since more than 50% of all the proteins from our dataset belong to the N.C. group, we checked the reliability of the obtained results by repeating the previous analyses on those organisms where the total lengths of proteins in the N.C. group are at most 20% of the total proteome length. The obtained results are different in range with respect to the complete dataset; however, all the relationships established above are conserved (Additional file 1: Figures S11 and S12). Bacteria still have higher disorder content than Archaea, plasmids have higher disorder content than chromosomes, and N.C. proteins have a higher disorder level than other COG groups (Additional file 1: Figure S13).

### Disorder content of proteins of specific function (toxins and antitoxins)

One specific class of plasmid-encoded proteins with known functions are toxin/antitoxin proteins, which participate in a wide range of cellular events. We applied the IDP analysis of plasmid/chromosomes as well as COG groups and categories to toxin/antitoxin proteins in order to support the findings relating disorder content with protein function (rather than its gene location). Because of the known involvement of structural disorder in protein function [7], we analyzed 11,564 Type II toxin/antitoxin proteins for: (a) abundance in disorder content and (b) their presence in chromosomes and plasmids. We chose the Type II toxin/antitoxin group as it is among the best described in the literature and because both toxins and antitoxins have a proteinaceous nature. We primarily considered the results obtained on Bacterial toxin/antitoxin proteins because of the small number of Archaeal proteins. Since toxins and antitoxins are relatively short proteins (their length is below 200 AA, with a few exceptions), we present the percentages of disordered AAs as a measure of disorder. The results are presented for the disorder predictor IsUnstruct only, because it is more appropriate for short proteins and give the most consistent results.

**Fig. 11:**
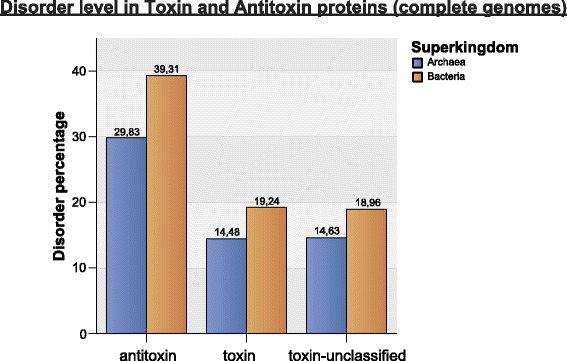
Disorder level in Toxin and Antitoxin proteins (complete genomes)

**Fig. 12:**
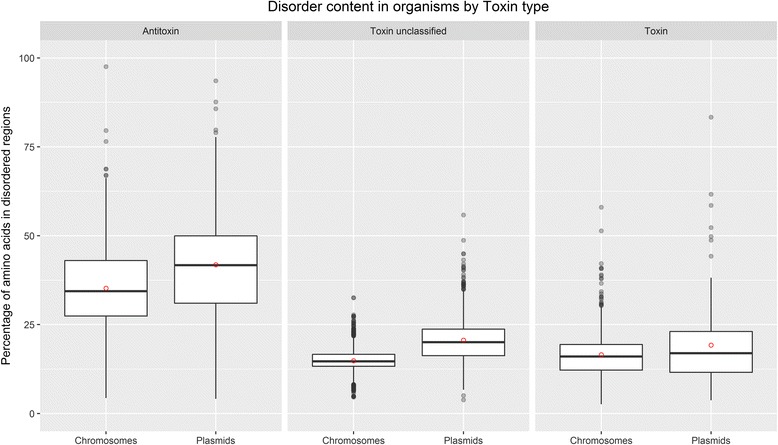
Disorder contents of chromosome- and plasmid-encoded toxin, antitoxin and toxin-unclassified proteins. The disorder content represents the percentage of amino acids in disordered regions, predicted by the IsUnstruct predictor

As can be seen in Table 2 and Fig. 11, the antitoxin proteins in comparison to toxin and toxin-unclassified proteins (proteins from our database that are not present in toxin/antitoxin database), are the most disordered (almost double) in both Archaeal and Bacterial proteomes. Antitoxin proteins are about one-third of the toxin-unclassified protein length and slightly shorter than toxin proteins. In Bacteria, the disorder content in antitoxin proteins encoded by plasmids is 17.6% higher than in chromosome-encoded antitoxin proteins (42.18% / 35.86% = 1.176), whereas in toxin proteins the disorder contents are almost equal (Fig. 12).

**Fig. 13:**
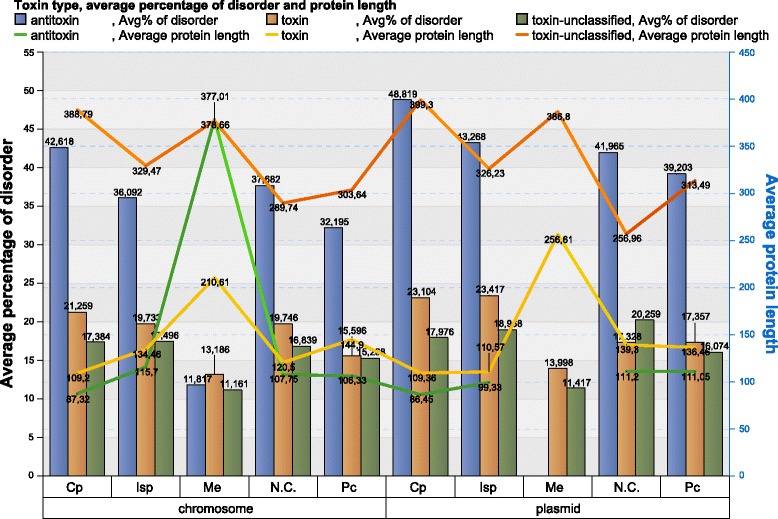
Disorder contents of chromosome- and plasmid-encoded toxin, antitoxin and toxin-unclassified proteins according to COG groups for Bacteria. For Arcahea and exact data, see Additional file 1: Figure S16

Bacterial proteins in the Me group have the lowest and almost equal disorder contents, regardless of the group to which they belong (antitoxin/toxin/toxin-unclassified) and source (chromosome−/plasmid-encoded). The disorder level in other COG groups (Cp, Isp, N.C. and Pc) is higher in antitoxins than in toxins or toxin-unclassified proteins. Also, the disorder level in Cp, Isp, N.C. and Pc plasmid-encoded proteins is higher than in all groups of chromosome-encoded proteins (antitoxin, toxin and toxin-unclassified) (Fig. 13).

Previous analyses of the disorder contents of toxin/antitoxin proteins was focused on the role of intrinsic disorder in the functioning and regulation of Type II toxin/antitoxin systems [7]. Type II toxins function by inhibiting either replication or translation [8]. Antitoxin proteins usually consist of a DNA-binding domain and a toxin-binding domain. The toxin-binding domain is usually a C-terminal IDP region that folds upon binding to a toxin. This domain is also important for their turnover, i.e. susceptibility to proteolytic degradation. Less is known about the disorder content in toxins and its role.

Our results are in accordance with previous results regarding the high disorder content and short protein length of antitoxin proteins [40] and their high susceptibility to proteolytic degradation, whereas their cognate toxins are comparatively stable [8, 41]. The results for the toxin/antitoxin proteins suggest that the protein function has stronger influence on disorder content than the protein gene location (on chromosome or plasmid).

## Conclusion

In this paper we analyzed the disorder content in prokaryotic plasmid-encoded proteins. The analysis was performed using three predictors and three measures. All three predictors and all three measures gave highly correlated results. The obtained results revealed that: (1) Bacteria exhibit significantly more disorder than Archaea. (2) Plasmid-encoded proteins have significantly higher disorder content than chromosome-encoded proteins in both prokaryote superkingdoms. (3) Classification according to COGs revealed that (a) proteins belonging to the metabolic group have a significantly lower disorder content than proteins in other groups, and that (b) plasmid-encoded proteins have a significantly higher disorder content only in the Not in COG group (where most of them are annotated as hypothetical proteins) as compared to chromosome-encoded proteins. (4) The analysis of antitoxin and toxin proteins (Type II) showed that (a) antitoxin proteins (both plasmid- and chromosome-encoded) contain much higher (almost double) disorder content than either toxin or toxin-unclassified proteins; (b) the disorder content in plasmid-encoded antitoxin proteins is higher than in respective chromosome-encoded proteins; (c) the disorder content in plasmid-encoded toxin proteins is almost the same as in respective chromosome-encoded proteins; (d) Bacterial proteins in the metabolic group have the lowest disorder content among COG groups; the disorder content is almost not dependent on group (antitoxin/toxin/toxin-unclassified) or source (chromosome−/plasmid-encoded).

Plasmids harbour lots of hypothetical proteins, many of these likely being products of ORFan genes and thus being relatively new in evolutionary terms. These may contribute to the improved adaptability of the organism by accommodating adaptive changes within short time frames, a role for which structurally disordered regions are highly suited. Our results suggest that while disorder content depends on genome and proteome characteristics, it is more influenced by functional engagements than by gene location (on chromosome or plasmid). Therefore, plasmid-encoded proteins are more disordered on average because a larger fraction of them fulfill functions that rely on structural disorder.

## Additional file

Additional file 1: This file includes additional tables and figures not shown in the manuscript. (ZIP 6200 kb)

## **Abbreviations**

**AA**: **Amino acid**
**COGs**: **Clusters of Orthologous Groups**
**Cp**: **Cellular processes proteins**
**IDPs**: **Intrinsically disordered proteins**
**IDRs**: **Intrinsically disordered (protein) regions**
**Isp**: **Information storage and processing proteins**
**Me**: **Metabolism proteins**
**N.C.**: **Proteins Not in COGs**
**Pc**: **Poorly characterized proteins**
