# Supplementary material for: Structural disorder of plasmid-encoded proteins in Bacteria and Archaea
Source: BMC Bioinformatics. 2018 Apr 25;19:158. doi: 10.1186/s12859-018-2158-6 (PMC5922023; doi:10.1186/s12859-018-2158-6)
Supplement: Supplementary file 1 — This file includes additional tables and figures not shown in the manuscript. (ZIP 6200 kb) [file 12859_2018_2158_MOESM1_ESM.zip › Supplementary/s.figure10/s.figure_10._archaea_disorder_content_hyp_nonhyp_cog.pdf]

Disorder content in hypothetical proteins in comparison to non-hypothetical proteins for Archaea

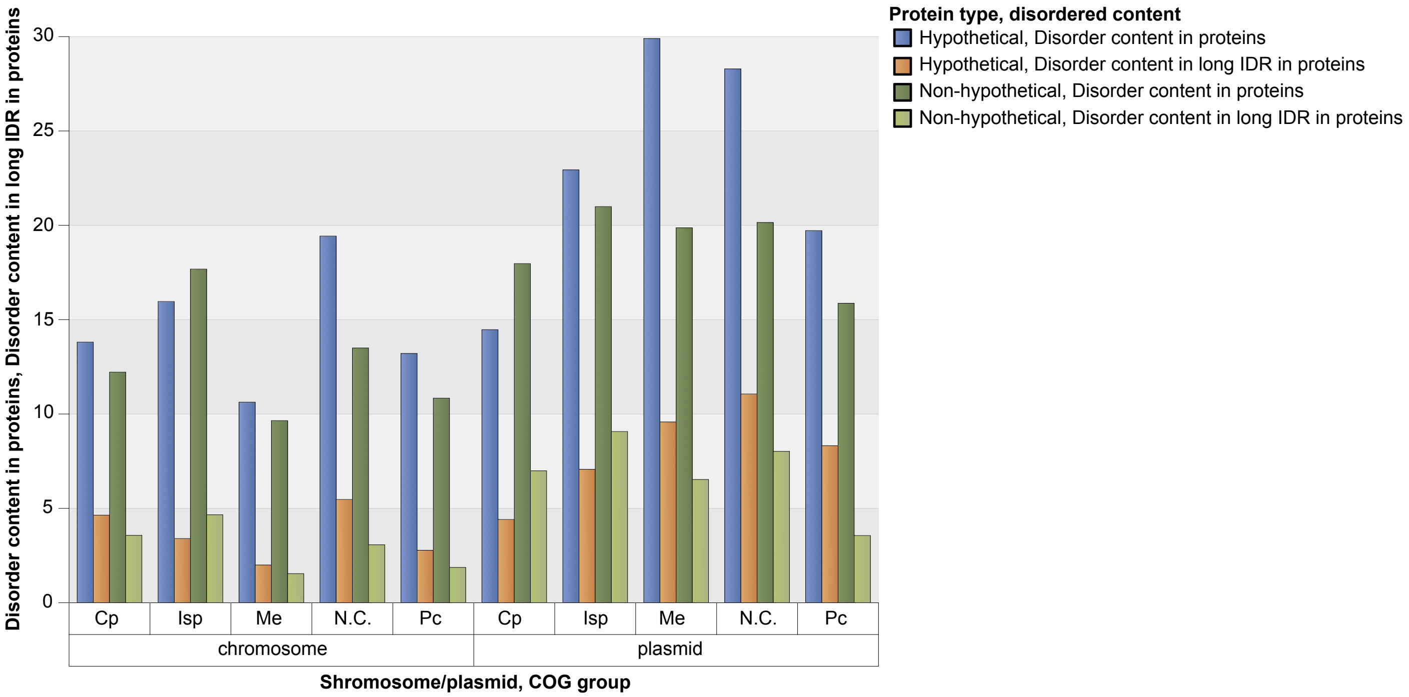

		Hypothetical		Non-hypothetical	
		% of disorderd AA	% of disorderd AA in long dis. reg.	% of disorderd AA	% of disorderd AA in long dis. reg.
chromosome	Cp	13.81	4.64	12.22	3.57
	Isp	15.96	3.4	17.68	4.66
	Me	10.63	2	9.65	1.54
	N.C.	19.43	5.47	13.5	3.07
	Pc	13.21	2.78	10.84	1.87
plasmid	Cp	14.47	4.41	17.97	6.99
	Isp	22.94	7.07	20.99	9.07
	Me	29.9	9.58	19.87	6.53
	N.C.	28.29	11.06	20.15	8.02
	Pc	19.72	8.32	15.87	3.56
