# Supplementary material for: Structural disorder of plasmid-encoded proteins in Bacteria and Archaea
Source: BMC Bioinformatics. 2018 Apr 25;19:158. doi: 10.1186/s12859-018-2158-6 (PMC5922023; doi:10.1186/s12859-018-2158-6)
Supplement: Supplementary file 1 — This file includes additional tables and figures not shown in the manuscript. (ZIP 6200 kb) [file 12859_2018_2158_MOESM1_ESM.zip › Supplementary/s.figure10/s.figure_10._bacteria_disorder_content_hyp_nonhyp_cog.pdf]

Disorder content in hypothetical proteins in comparison to non-hypothetical proteins for Bacteria

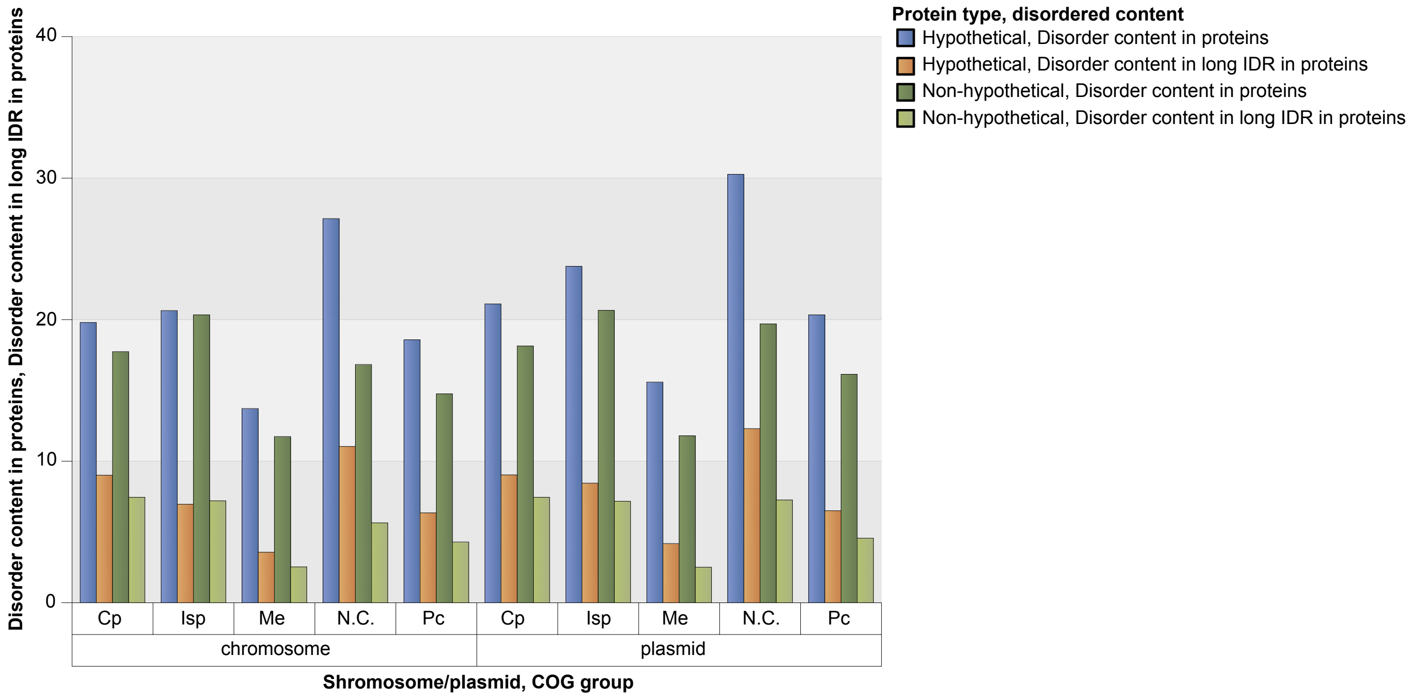

		Hypothetical		Non-hypothetical	
		% of disorderd AA	% of disorderd AA in long dis. reg.	% of disorderd AA	% of disorderd AA in long dis. reg.
chromosome	Cp	19.8	9.01	17.74	7.45
	Isp	20.64	6.96	20.34	7.2
	Me	13.72	3.57	11.74	2.53
	N.C.	27.14	11.04	16.83	5.64
	Pc	18.58	6.35	14.76	4.29
plasmid	Cp	21.11	9.03	18.14	7.45
	Isp	23.77	8.45	20.66	7.17
	Me	15.59	4.18	11.8	2.51
	N.C.	30.27	12.3	19.7	7.26
	Pc	20.34	6.5	16.14	4.56
