# Supplementary material for: Structural disorder of plasmid-encoded proteins in Bacteria and Archaea
Source: BMC Bioinformatics. 2018 Apr 25;19:158. doi: 10.1186/s12859-018-2158-6 (PMC5922023; doi:10.1186/s12859-018-2158-6)

Disorder content in long(>30) IDR in proteins in different data subsets for Bacteria where genomes with at most 20% of their proteins belonging to the N.C. group

SUPERKINGDOM: Bacteria

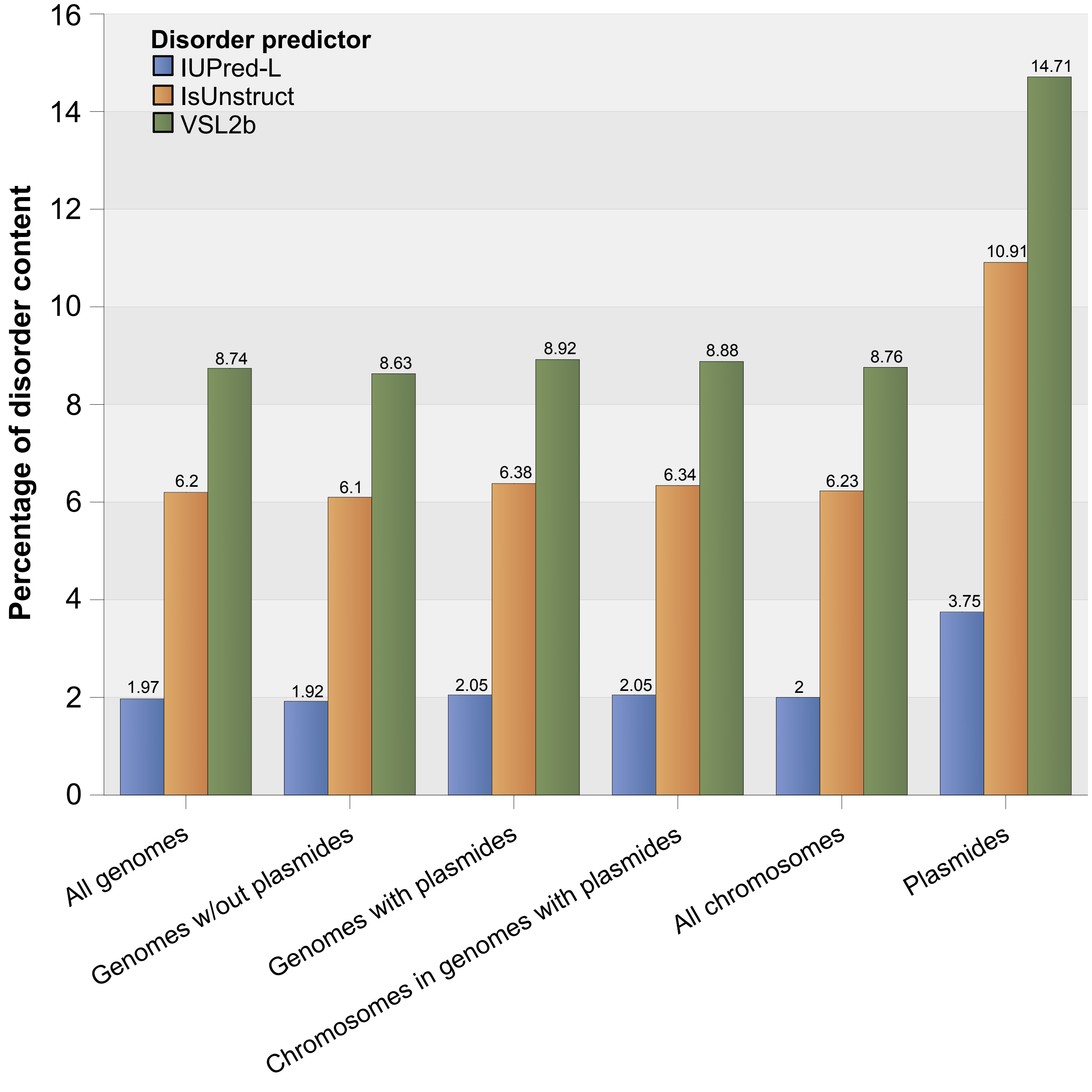

Supplement: Supplementary file 1 — This file includes additional tables and figures not shown in the manuscript. (ZIP 6200 kb) [file 12859_2018_2158_MOESM1_ESM.zip › Supplementary/s.figure12/s.figure_12._bacteria_20_dis_31.pdf]
