# Supplementary material for: Structural disorder of plasmid-encoded proteins in Bacteria and Archaea
Source: BMC Bioinformatics. 2018 Apr 25;19:158. doi: 10.1186/s12859-018-2158-6 (PMC5922023; doi:10.1186/s12859-018-2158-6)
Supplement: Supplementary file 1 — This file includes additional tables and figures not shown in the manuscript. (ZIP 6200 kb) [file 12859_2018_2158_MOESM1_ESM.zip › Supplementary/s.figure13/s.figure_13.bacteria_80_dis.pdf]

Disorder content of different COG groups and data subsets for organisms where genomes with at most 20% of their proteins belonging to the N.C. group for Bacteria

SUPERKINGDOM: Bacteria

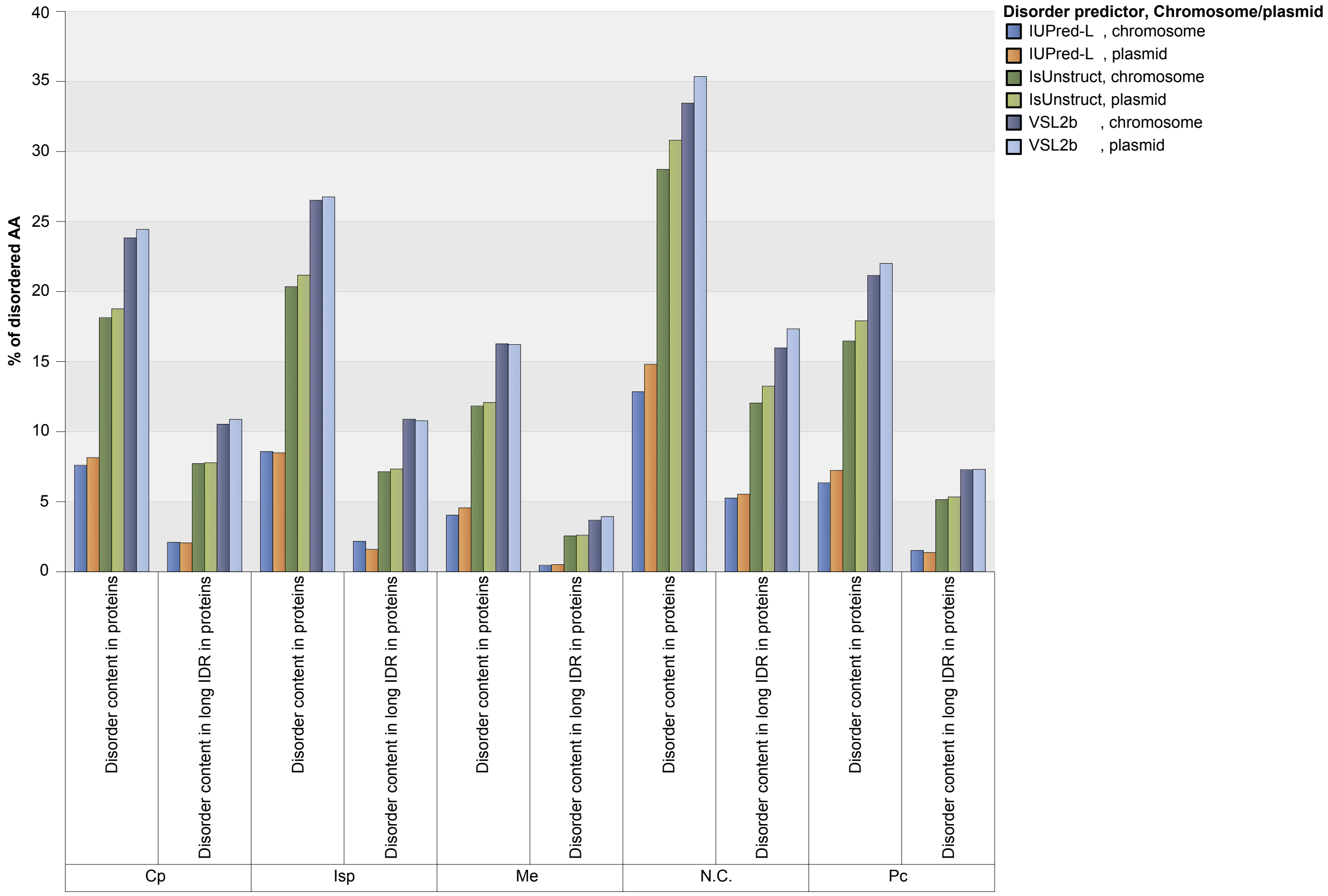

		IUPred-L		IsUnstruct		VSL2b	
		chromosome	plasmid	chromosome	plasmid	chromosome	plasmid
Cp	Disorder content in proteins	7.6	8.15	18.14	18.77	23.83	24.44
	Disorder content in long IDR in proteins	2.1	2.06	7.72	7.78	10.53	10.88
Isp	Disorder content in proteins	8.58	8.49	20.35	21.17	26.52	26.76
	Disorder content in long IDR in proteins	2.17	1.6	7.14	7.33	10.89	10.78
Me	Disorder content in proteins	4.04	4.56	11.83	12.08	16.27	16.22
	Disorder content in long IDR in proteins	0.46	0.51	2.56	2.61	3.68	3.94
N.C.	Disorder content in proteins	12.85	14.81	28.72	30.8	33.45	35.35
	Disorder content in long IDR in proteins	5.26	5.53	12.04	13.25	15.98	17.34
Pc	Disorder content in proteins	6.35	7.23	16.47	17.91	21.15	22.01
	Disorder content in long IDR in proteins	1.52	1.37	5.15	5.34	7.29	7.31
