# Supplementary material for: Structural disorder of plasmid-encoded proteins in Bacteria and Archaea
Source: BMC Bioinformatics. 2018 Apr 25;19:158. doi: 10.1186/s12859-018-2158-6 (PMC5922023; doi:10.1186/s12859-018-2158-6)
Supplement: Supplementary file 1 — This file includes additional tables and figures not shown in the manuscript. (ZIP 6200 kb) [file 12859_2018_2158_MOESM1_ESM.zip › Supplementary/s.figure16/s.figure_16.avg_dis_len_cog_archaea.pdf]

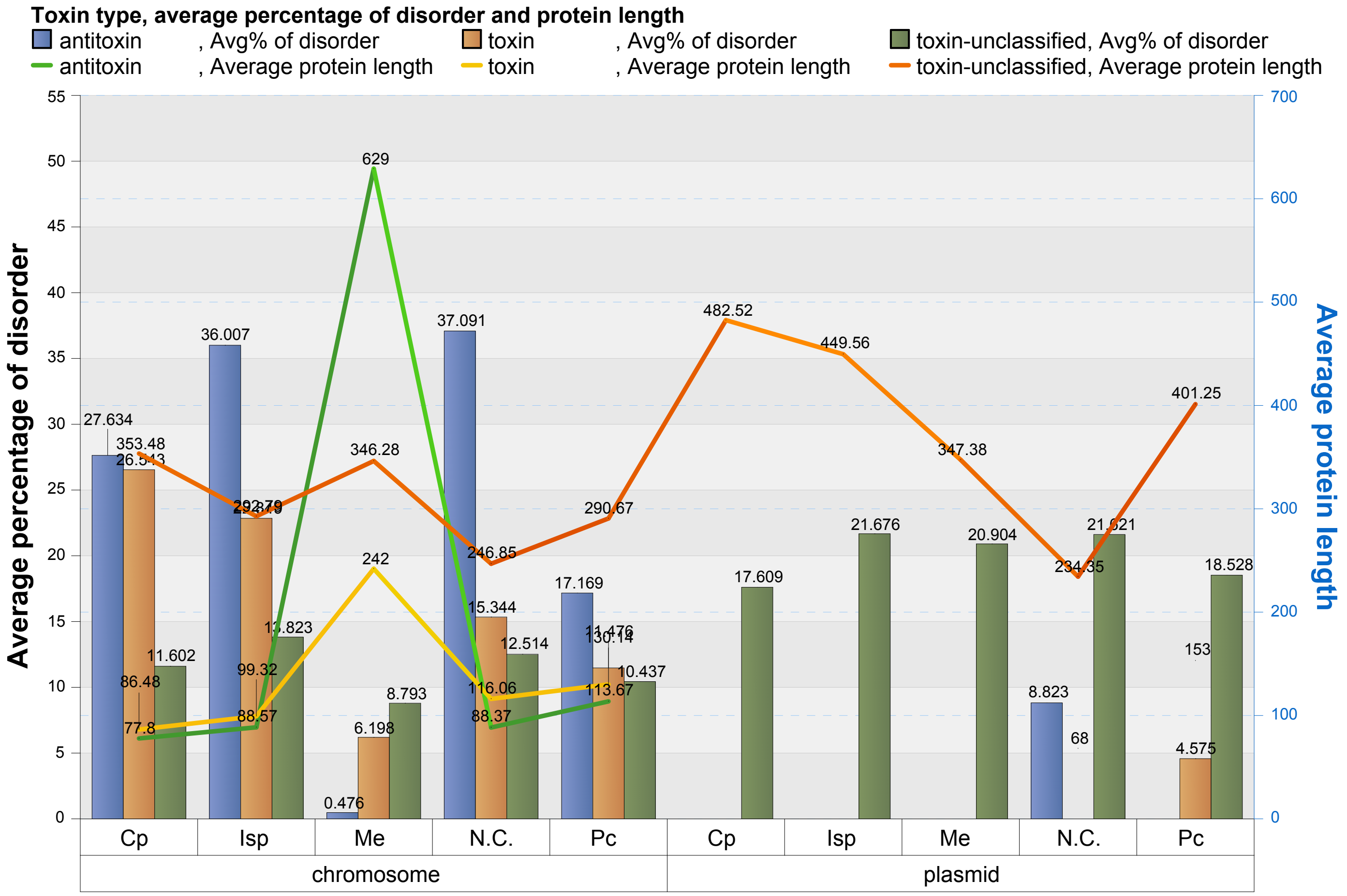

			antitoxin			toxin			toxin-unclassified		
			Average protein length	Average % of protein disorder	Number of proteins	Average protein length	Average % of protein disorder	Number of proteins	Average protein length	Average % of protein disorder	Number of proteins
Archaea	chromosome	Cp	77.8	27.634	10	86.48	26.543	47	353.48	11.602	23,786
		Isp	88.57	36.007	47	99.32	22.849	46	292.79	13.823	35,181
		Me	629	0.476	1	242	6.198	1	346.28	8.793	65,161
		N.C.	88.37	37.091	218	116.06	15.344	126	246.85	12.514	137,096
		Pc	113.67	17.169	243	130.14	11.476	328	290.67	10.437	43,957
	plasmid	Cp							482.52	17.609	100
		Isp							449.56	21.676	198
		Me							347.38	20.904	55
		N.C.	68	8.823	1				234.35	21.621	1,042
		Pc				153	4.575	1	401.25	18.528	98
