# Supplementary material for: Structural disorder of plasmid-encoded proteins in Bacteria and Archaea
Source: BMC Bioinformatics. 2018 Apr 25;19:158. doi: 10.1186/s12859-018-2158-6 (PMC5922023; doi:10.1186/s12859-018-2158-6)
Supplement: Supplementary file 1 — This file includes additional tables and figures not shown in the manuscript. (ZIP 6200 kb) [file 12859_2018_2158_MOESM1_ESM.zip › Supplementary/s.figure16/s.figure_16.avg_dis_len_cog_bacteria.pdf]

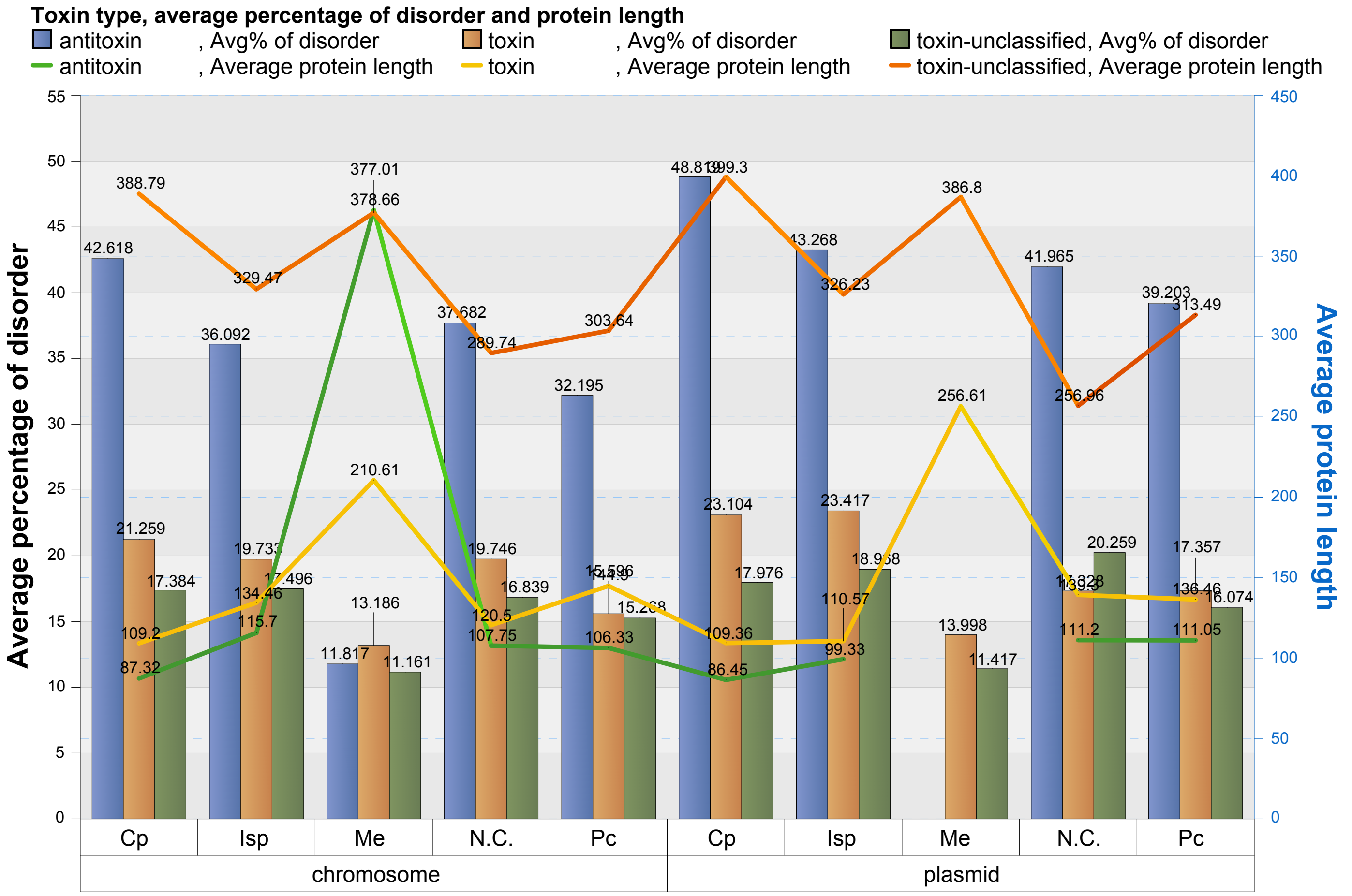

			antitoxin			toxin			toxin-unclassified		
			Average protein length	Average % of protein disorder	Number of proteins	Average protein length	Average % of protein disorder	Number of proteins	Average protein length	Average % of protein disorder	Number of proteins
Bacteria	chromosome	Cp	87.32	42.618	481	109.2	21.259	566	388.79	17.384	815,045
		Isp	115.7	36.092	1,493	134.46	19.733	217	329.47	17.496	695,033
		Me	378.66	11.817	12	210.61	13.186	114	377.01	11.161	1,435,900
		N.C.	107.75	37.682	1,549	120.5	19.746	1,021	289.74	16.839	4,584,662
		Pc	106.33	32.195	1,336	144.9	15.596	3,127	303.64	15.268	738,635
	plasmid	Cp	86.45	48.819	48	109.36	23.104	41	399.3	17.976	17,210
		Isp	99.33	43.268	99	110.57	23.417	19	326.23	18.968	19,305
		Me				256.61	13.998	13	386.8	11.417	29,551
		N.C.	111.2	41.965	130	139.3	17.328	86	256.96	20.259	165,363
		Pc	111.05	39.203	106	136.46	17.357	243	313.49	16.074	15,407
