# Supplementary material for: Structural disorder of plasmid-encoded proteins in Bacteria and Archaea
Source: BMC Bioinformatics. 2018 Apr 25;19:158. doi: 10.1186/s12859-018-2158-6 (PMC5922023; doi:10.1186/s12859-018-2158-6)
Supplement: Supplementary file 1 — This file includes additional tables and figures not shown in the manuscript. (ZIP 6200 kb) [file 12859_2018_2158_MOESM1_ESM.zip › Supplementary/s.figure3/s.figure_3._archaea_cog_percent_prot.pdf]

Percentage of proteins in COG categories for Archaea

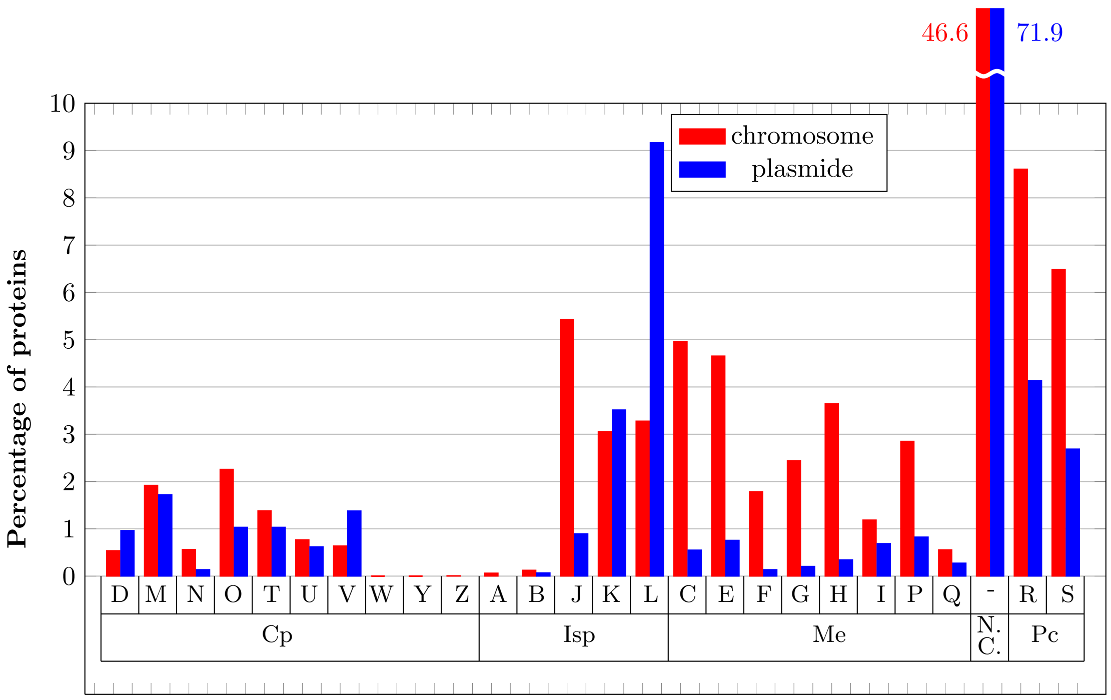

COG group/category

		Chromosome		Plasmid	
Group	Category	% of proteins	Number of proteins	% of proteins	Number of proteins
Cp	D	0.53883	1590	0.96485	14
	M	1.92047	5667	1.72294	25
	N	0.56323	1662	0.13783	2
	O	2.25936	6667	1.03376	15
	T	1.38198	4078	1.03376	15
	U	0.76893	2269	0.62026	9
	V	0.63778	1882	1.37835	20
	W	0.00033	1	—	—
	Y	0.00033	1	—	—
	Z	0.00881	26	—	—
Isp	A	0.06404	189	—	—
	B	0.12538	370	0.06891	1
	J	5.42694	16014	0.89593	13
	K	3.05880	9026	3.51481	51
	L	3.27873	9675	9.16609	133
Me	C	4.95589	14624	0.55134	8
	E	4.65530	13737	0.75809	11
	F	1.78729	5274	0.13783	2
	G	2.44338	7210	0.20675	3
	H	3.64575	10758	0.34458	5
	I	1.18712	3503	0.68917	10
	P	2.85174	8415	0.82701	12
	Q	0.55645	1642	0.27567	4
N.C.	-	46.57672	137440	71.88146	1043
Pc	R	8.60740	25399	4.13507	60
	S	6.48258	19129	2.68780	39
