# Supplementary material for: Structural disorder of plasmid-encoded proteins in Bacteria and Archaea
Source: BMC Bioinformatics. 2018 Apr 25;19:158. doi: 10.1186/s12859-018-2158-6 (PMC5922023; doi:10.1186/s12859-018-2158-6)
Supplement: Supplementary file 1 — This file includes additional tables and figures not shown in the manuscript. (ZIP 6200 kb) [file 12859_2018_2158_MOESM1_ESM.zip › Supplementary/s.figure3/s.figure_3._bacteria_cog_percent_prot.pdf]

Percentage of proteins in COG categories for Bacteria

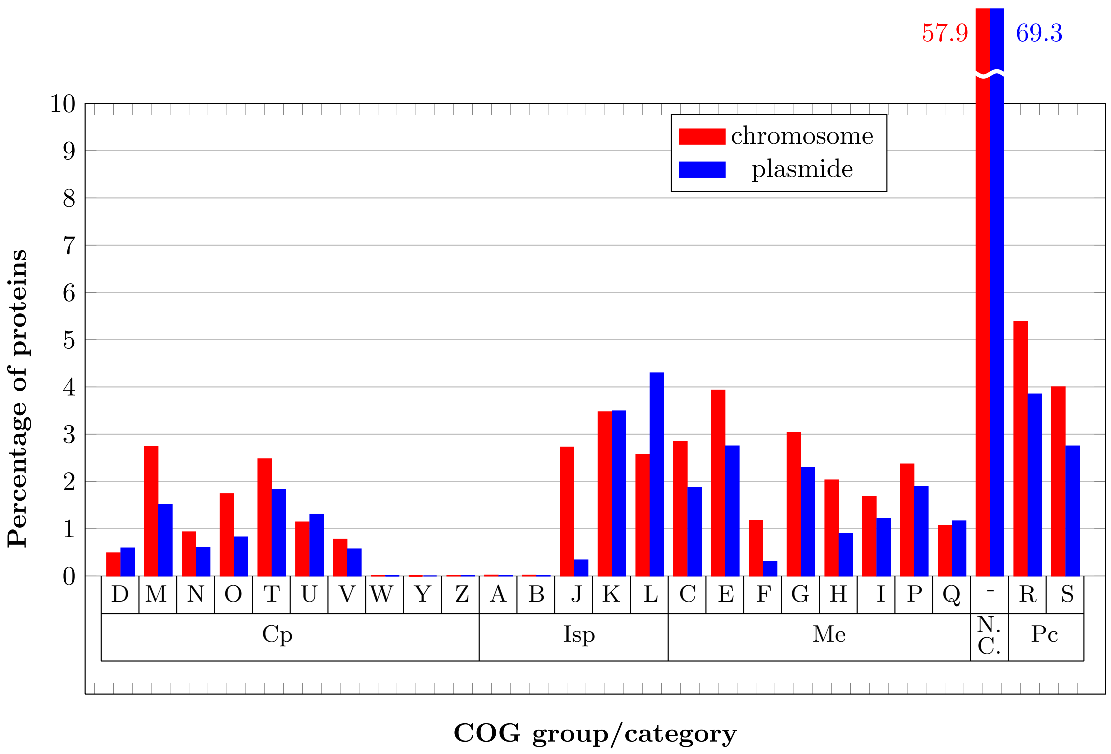

		Chromosome		Plasmid	
Group	Category	% of proteins	Number of proteins	% of proteins	Number of proteins
Cp	D	0.48692	38564	0.59046	1410
	M	2.74218	217177	1.51511	3618
	N	0.93107	73740	0.60679	1449
	O	1.73816	137660	0.82372	1967
	T	2.47731	196200	1.82416	4356
	U	1.14089	90357	1.30530	3117
	V	0.77686	61527	0.57036	1362
	Y	0.00003	3	—	—
	W	0.00417	331	0.00293	7
	Z	0.00672	533	0.00544	13
Isp	A	0.01650	1307	0.00502	12
	B	0.01575	1248	0.00418	10
	J	2.72499	215816	0.33752	806
	K	3.47177	274960	3.49129	8337
	L	2.56837	203412	4.29575	10258
Me	C	2.84932	225663	1.87483	4477
	E	3.93059	311298	2.74923	6565
	F	1.16796	92501	0.30193	721
	G	3.03180	240115	2.29318	5476
	H	2.03057	160819	0.89240	2131
	I	1.68201	133213	1.21108	2892
	P	2.36823	187561	1.89410	4523
	Q	1.07143	84856	1.16376	2779
N.C.	-	57.92057	4587232	69.33968	165579
Pc	R	5.38243	426282	3.84934	9192
	S	4.00026	316816	2.74881	6564
