# Supplementary material for: Structural disorder of plasmid-encoded proteins in Bacteria and Archaea
Source: BMC Bioinformatics. 2018 Apr 25;19:158. doi: 10.1186/s12859-018-2158-6 (PMC5922023; doi:10.1186/s12859-018-2158-6)
Supplement: Supplementary file 1 — This file includes additional tables and figures not shown in the manuscript. (ZIP 6200 kb) [file 12859_2018_2158_MOESM1_ESM.zip › Supplementary/s.figure5/Sup.Fig.5.2.pdf]

Disorder content in long (>30AA) disordered regions in Archaea and Bacteria by gene location, as a function of proteome size.

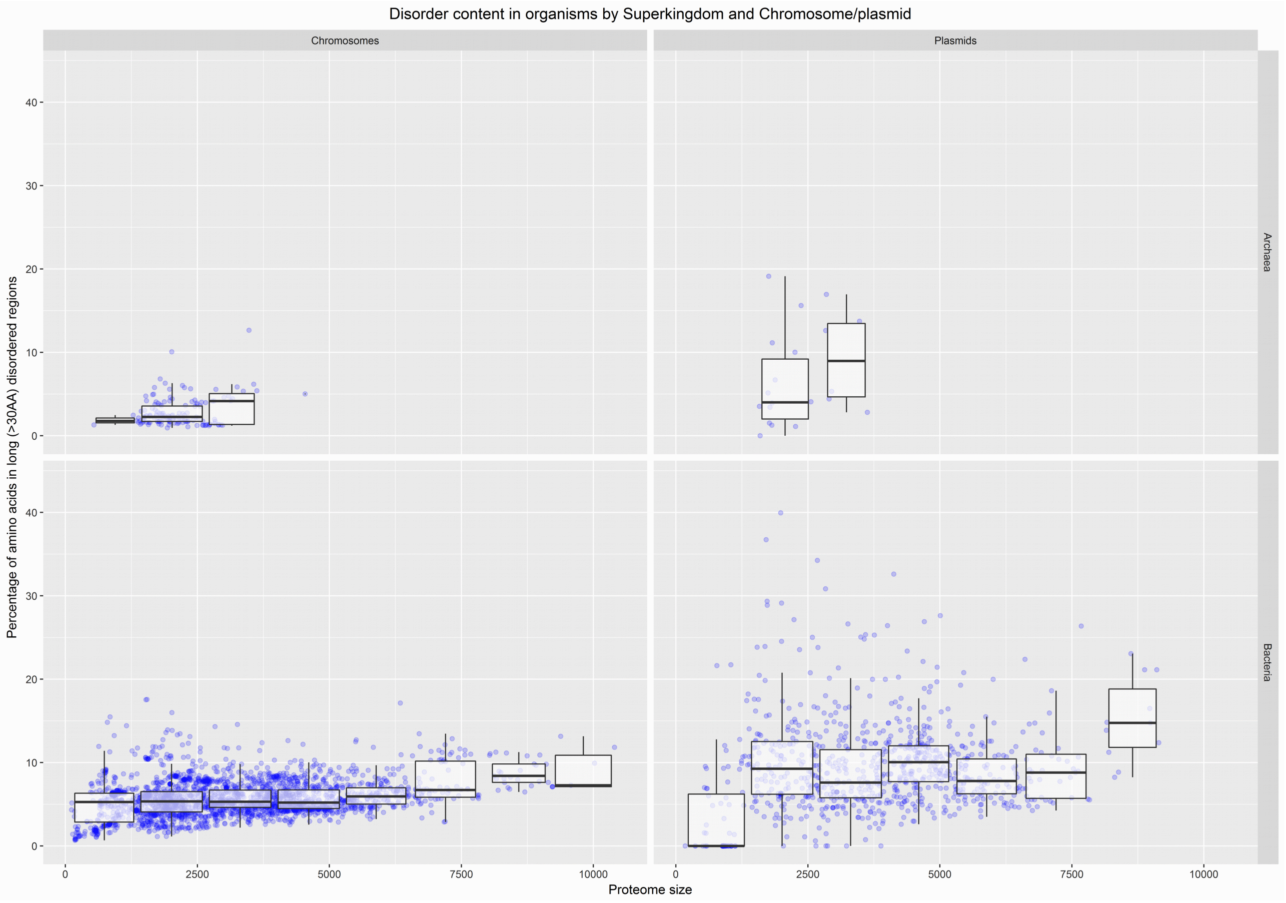

Disorder is predicted by the IsUnstruct predictor.

The box diagrams in the paper follow the usual representation:

1. the horizontal line inside a box represents the median value (50% of the samples is lower and 50% of the samples are higher than median);
2. lower box bound represents *first quartile* value (25% of data are lower and 75% are higher than first quartile);
3. upper box bound represents *third quartile* value (75% of data are lower and 25% are higher than third quartile);
4. the box height represents *interquartile range* (IQR); in the case of normal distribution, IQR = 1.35 x σ;
5. the whiskers (vertical lines above and under the box) ranges up to the highest datum within 1.5 x IQR of the upper quartile and down to the lowest datum within 1.5x IQR of the lower quartile;
6. the dots above the top whisker and under the bottom whisker represent outliers, i.e. the samples that are out of the range (in some of the diagrams each sample is represented as a dot, and outliers are not specifically highlighted, because it is obvious which samples lay out of the whiskers range);
7. in some of the diagrams the red dot represents the mean value.
