# Supplementary material for: Structural disorder of plasmid-encoded proteins in Bacteria and Archaea
Source: BMC Bioinformatics. 2018 Apr 25;19:158. doi: 10.1186/s12859-018-2158-6 (PMC5922023; doi:10.1186/s12859-018-2158-6)
Supplement: Supplementary file 1 — This file includes additional tables and figures not shown in the manuscript. (ZIP 6200 kb) [file 12859_2018_2158_MOESM1_ESM.zip › Supplementary/s.figure9/s.figure_9._archaea_perc_prot_dis_31.pdf]

Percentage of proteins in COG category with long(>30AA) disordered regions in Archaea

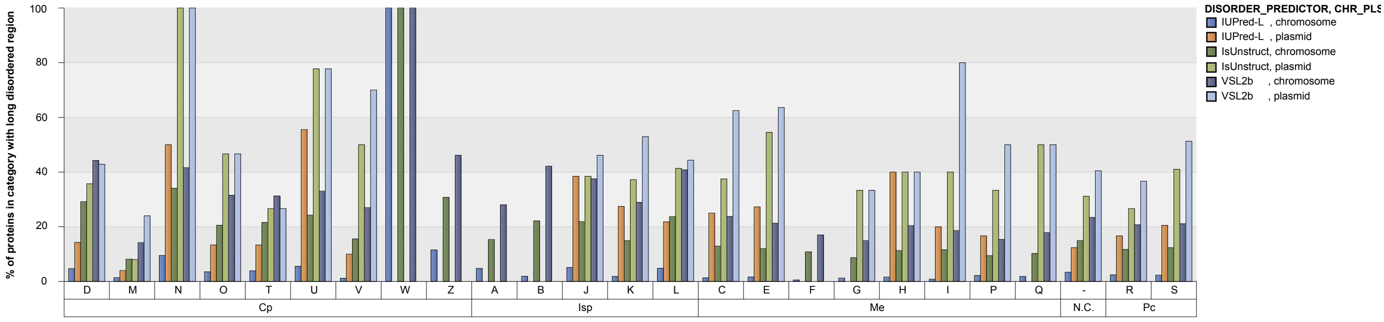

			Cp									Isp					Me								N.C.	Pc	
			D	M	N	O	T	U	V	W	Z	A	B	J	K	L	C	E	F	G	H	I	P	Q	-	R	S
IUPred-L	chromosome	Number of proteins	1,590	5,667	1,662	6,667	4,078	2,269	1,882	1	26	189	370	16,014	9,026	9,675	14,624	13,737	5,274	7,210	10,758	3,503	8,415	1,642	137,440	25,399	19,129
		% of AA in long disordered regions	0.94	0.23	1.48	0.65	0.57	1.08	0.15	37.25	2.32	2.27	0.2	1.5	0.4	0.74	0.23	0.18	0.09	0.16	0.21	0.18	0.25	0.27	1.17	0.45	0.5
		% of proteins with long disordered region	4.71	1.41	9.5	3.52	3.87	5.55	1.16	100	11.53	4.76	1.89	5.12	1.83	4.81	1.36	1.63	0.54	1.24	1.61	0.85	2.18	1.82	3.39	2.42	2.33
	plasmid	Number of proteins	14	25	2	15	15	9	20					13	51	133	8	11			5	10	12		1,043	60	39
		% of AA in long disordered regions	2.93	0.18	22.83	1.3	1.73	5.21	1.16					7.47	4.74	3.55	2.7	4.12			4.88	2.73	3.17		4.41	3.72	5.36
		% of proteins with long disordered region	14.28	4	50	13.33	13.33	55.55	10					38.46	27.45	21.8	25	27.27			40	20	16.66		12.36	16.66	20.51
IsUnstruct	chromosome	Number of proteins	1,590	5,667	1,662	6,667	4,078	2,269	1,882	1	26	189	370	16,014	9,026	9,675	14,624	13,737	5,274	7,210	10,758	3,503	8,415	1,642	137,440	25,399	19,129
		% of AA in long disordered regions	7.45	1.16	7.53	4.19	3.55	5.36	2.16	57.51	7.46	3.82	8.77	5.5	3.61	3.53	2.09	1.44	1.72	1	1.63	1.49	1.31	1.34	4.48	1.88	2.92
		% of proteins with long disordered region	29.18	8.17	34.11	20.56	21.57	24.28	15.56	100	30.76	15.34	22.16	21.88	14.91	23.7	12.91	12.04	10.82	8.71	11.31	11.59	9.44	10.23	14.93	11.72	12.36
	plasmid	Number of proteins	14	25	2	15	15	9	20					13	51	133	8	11		3	5	10	12	4	1,043	60	39
		% of AA in long disordered regions	13.64	0.84	27.51	7.87	4.22	11.26	5.52					10.84	10.93	7.82	4.85	10.96		2.99	12.32	4.09	7.74	4.97	10.59	4.64	9.46
		% of proteins with long disordered region	35.71	8	100	46.66	26.66	77.77	50					38.46	37.25	41.35	37.5	54.54		33.33	40	40	33.33	50	31.16	26.66	41.02
VSL2b	chromosome	Number of proteins	1,590	5,667	1,662	6,667	4,078	2,269	1,882	1	26	189	370	16,014	9,026	9,675	14,624	13,737	5,274	7,210	10,758	3,503	8,415	1,642	137,440	25,399	19,129
		% of AA in long disordered regions	12.91	2.17	10.97	7.72	5.84	8.22	4.04	76.47	10.61	6.52	18.44	11.79	8.35	7.67	4.35	3	2.67	1.88	3.25	2.78	2.44	2.58	7.65	3.78	5.65
		% of proteins with long disordered region	44.27	14.22	41.63	31.52	31.26	33.05	26.99	100	46.15	28.04	42.16	37.52	28.92	40.8	23.78	21.33	17	14.97	20.44	18.61	15.37	17.84	23.4	20.71	21.1
	plasmid	Number of proteins	14	25	2	15	15	9	20					13	51	133	8	11		3	5	10	12	4	1,043	60	39
		% of AA in long disordered regions	16.61	3.26	37.4	10.19	7.85	12.45	9.3					14.55	17.38	10.1	8.95	19.73		2.84	15.55	17.48	13.65	6.36	15.02	7.82	14.1
		% of proteins with long disordered region	42.85	24	100	46.66	26.66	77.77	70					46.15	52.94	44.36	62.5	63.63		33.33	40	80	50	50	40.46	36.66	51.28
