# Supplementary material for: Structural disorder of plasmid-encoded proteins in Bacteria and Archaea
Source: BMC Bioinformatics. 2018 Apr 25;19:158. doi: 10.1186/s12859-018-2158-6 (PMC5922023; doi:10.1186/s12859-018-2158-6)
Supplement: Supplementary file 1 — This file includes additional tables and figures not shown in the manuscript. (ZIP 6200 kb) [file 12859_2018_2158_MOESM1_ESM.zip › Supplementary/s.figure_1.average_protein_number_and_length_in_data_subsets.pdf]

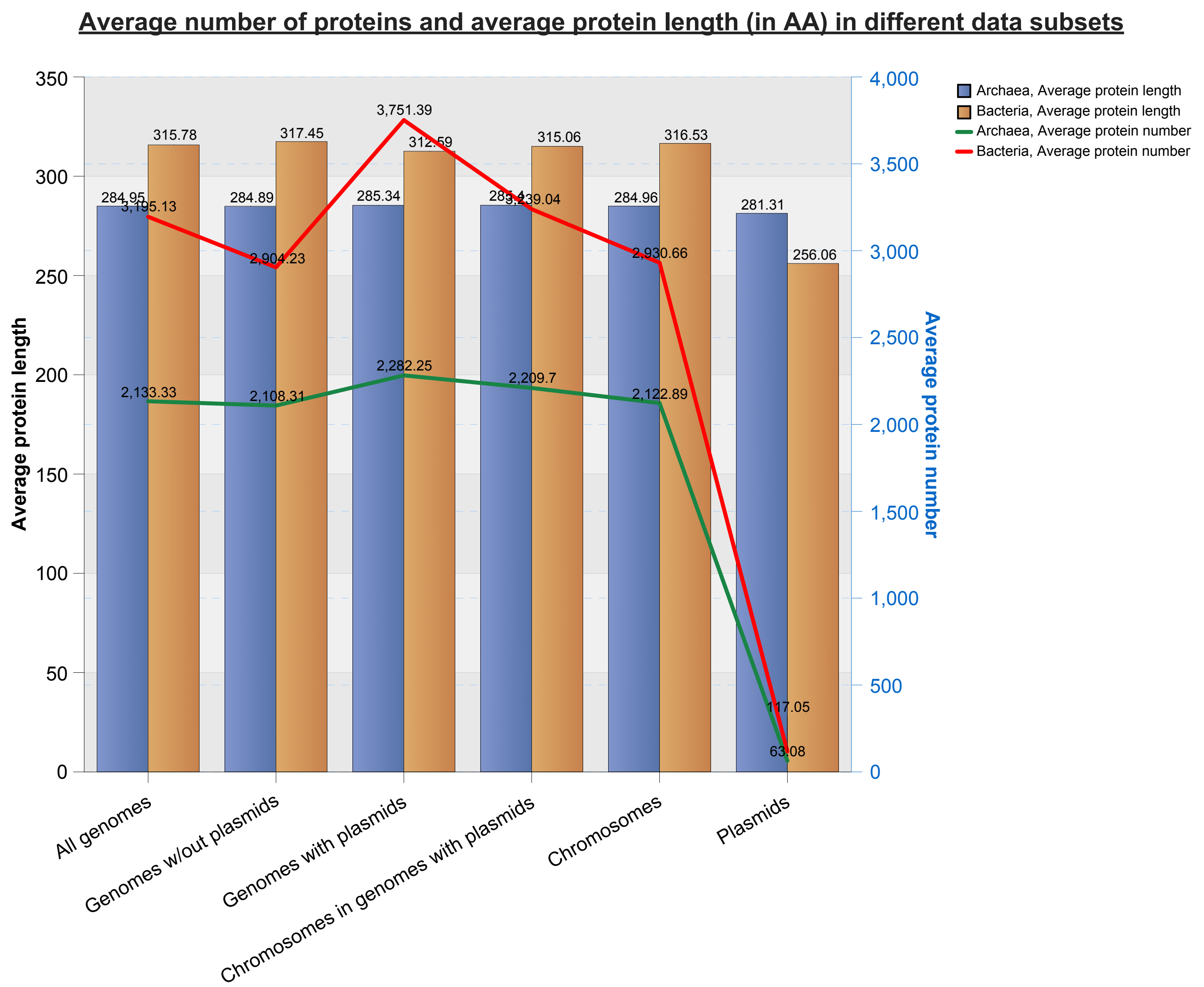

Average protein length is rather uniform in different types of Archaeal material – between 284 (in Archaeal chromosomes, genomes without plasmids and complete genomes) and 286 AA (either in complete genomes that includes plasmids either in chromosomes that belongs to genomes with plasmids). Average protein length in Archaeal plasmids is somewhat shorter, but only for a few AA – 281AA. Average protein length is higher in Bacteria material (between 312 and 318AA) except in plasmids where it is much shorter (around 256AA).
