# Supplementary material for: Structural disorder of plasmid-encoded proteins in Bacteria and Archaea
Source: BMC Bioinformatics. 2018 Apr 25;19:158. doi: 10.1186/s12859-018-2158-6 (PMC5922023; doi:10.1186/s12859-018-2158-6)
Supplement: Supplementary file 1 — This file includes additional tables and figures not shown in the manuscript. (ZIP 6200 kb) [file 12859_2018_2158_MOESM1_ESM.zip › Supplementary/s.figure_14.toxin_antitoxin_avg_disorder.pdf]

# Average disorder level and protein length of Toxin/Antitoxin proteins in chromosomes and plasmids

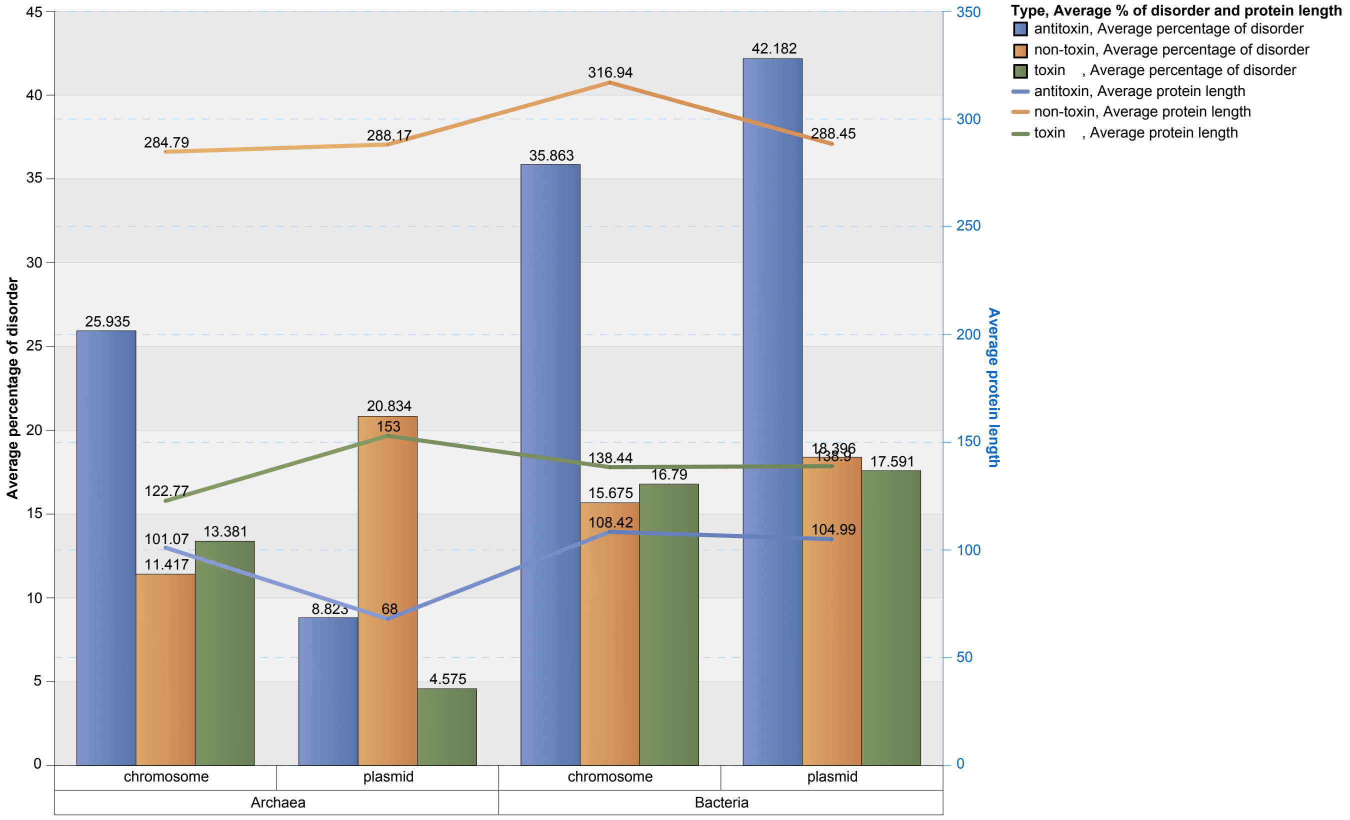


| | | antitoxin | | | non-toxin | | | toxin | | |
|---|---|---|---|---|---|---|---|---|---|---|
| | | Avg protein len. | #proteins | Avg% of disorder | Avg protein len. | #proteins | Avg% of disorder | Avg protein len. | #proteins | Avg% of disorder |
| Archaea | chromosome | 101.07 | 519 | 25.935 | 284.79 | 294,049 | 11.417 | 122.77 | 515 | 13.381 |
| | plasmid | 68 | 1 | 8.823 | 288.17 | 1,449 | 20.834 | 153 | 1 | 4.575 |
| Bacteria | chromosome | 108.42 | 4,868 | 35.863 | 316.94 | 7,910,106 | 15.675 | 138.44 | 4,892 | 16.79 |
| | plasmid | 104.99 | 383 | 42.182 | 288.45 | 238,026 | 18.396 | 138.9 | 385 | 17.591 |

Predictor: IsUnstruct; Measure: number of disordered AA in proteins
