# Supplementary material for: Structural disorder of plasmid-encoded proteins in Bacteria and Archaea
Source: BMC Bioinformatics. 2018 Apr 25;19:158. doi: 10.1186/s12859-018-2158-6 (PMC5922023; doi:10.1186/s12859-018-2158-6)
Supplement: Supplementary file 1 — This file includes additional tables and figures not shown in the manuscript. (ZIP 6200 kb) [file 12859_2018_2158_MOESM1_ESM.zip › Supplementary/s.figure_7.protein_percent_in_cog_groups.pdf]

Protein percentage in COG groups over group of material

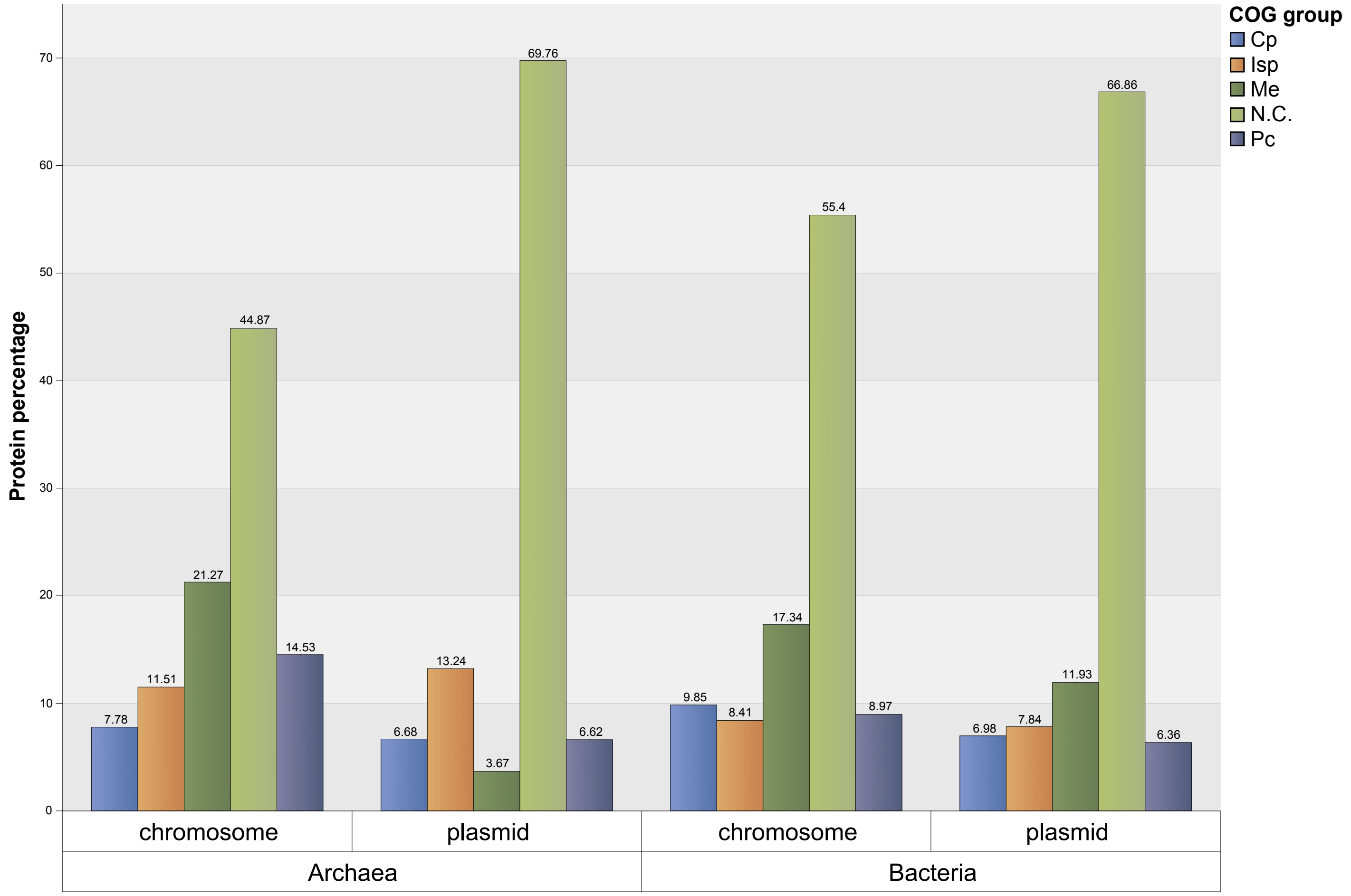

Protein percentage		Cp	Isp	Me	N.C.	Pc
Archaea	chromosome	7.78%	11.51%	21.27%	44.87%	14.53%
	plasmid	6.68%	13.24%	3.67%	69.76%	6.62%
Bacteria	chromosome	9.85%	8.41%	17.34%	55.40%	8.97%
	plasmid	6.98%	7.84%	11.93%	66.86%	6.36%
