# Supplementary material for: Structural disorder of plasmid-encoded proteins in Bacteria and Archaea
Source: BMC Bioinformatics. 2018 Apr 25;19:158. doi: 10.1186/s12859-018-2158-6 (PMC5922023; doi:10.1186/s12859-018-2158-6)
Supplement: Supplementary file 1 — This file includes additional tables and figures not shown in the manuscript. (ZIP 6200 kb) [file 12859_2018_2158_MOESM1_ESM.zip › Supplementary/s.table_1.Distribution_of_toxin_antitoxin_proteins.pdf]

Distribution of toxin/antitoxin proteins

PROTEIN_NUMBER			Cp	Isp	Me	N.C.	Pc	Total
Archaea	chromosome	antitoxin	10	47	1	218	243	**519**
		toxin	47	46	1	126	328	**548**
		**Total**	**57**	**93**	**2**	**344**	**571**	**1,067**
	plasmid	antitoxin				1		**1**
		toxin					1	**1**
		**Total**				**1**	**1**	**2**
	**Total**		**57**	**93**	**2**	**345**	**572**	**1,069**
Bacteria	chromosome	antitoxin	481	1,493	12	1,549	1,336	**4,871**
		toxin	566	217	114	1,021	3,127	**5,045**
		**Total**	**1,047**	**1,710**	**126**	**2,570**	**4,463**	**9,916**
	plasmid	antitoxin	48	99		130	106	**383**
		toxin	41	19	13	86	243	**402**
		**Total**	**89**	**118**	**13**	**216**	**349**	**785**
	**Total**		**1,136**	**1,828**	**139**	**2,786**	**4,812**	**10,701**
**Total**			**1,193**	**1,921**	**141**	**3,131**	**5,384**	**11,770**

Note: total number of proteins (11770) in COG groups are slightly higher than number of different proteins in the material (11564) because there are proteins assigned to more than one COG category: 33 in Archaea (toxin: 33 chromosome encoded) and 173 in Bacteria (antitoxin: 3 chromosome encoded and toxin: 153 chromosome encoded and 17 plasmid encoded proteins) . There are no proteins assigned to more that two categories.
