# Supplementary material for: Structural disorder of plasmid-encoded proteins in Bacteria and Archaea
Source: BMC Bioinformatics. 2018 Apr 25;19:158. doi: 10.1186/s12859-018-2158-6 (PMC5922023; doi:10.1186/s12859-018-2158-6)
Supplement: Supplementary file 1 — This file includes additional tables and figures not shown in the manuscript. (ZIP 6200 kb) [file 12859_2018_2158_MOESM1_ESM.zip › Supplementary/supplementary.html]

Plasmid-encoded protein structural disorder in Archaea and Bacteria


# Plasmid-encoded protein structural disorder in Archaea and Bacteria

## Supplementary data


---

## Tables

- #### Supplementary Table S1 - Distribution of toxin/antitoxin proteins
- #### Supplementary Table S2 - Organisms with at most 20% of their proteins belonging to the N.C. group
- #### Supplementary Table S3 - Correlation coefficients between different disorder measures for each predictor and both superkingdoms
- #### Supplementary Table S4 - Correlation coefficients between different predictors for each disorder measure and both superkingdoms

---

## Figures

- #### Supplementary figure Fig. S1 - Average number of proteins and average protein length (in AA) in different data subsets
- #### Supplementary figure Fig. S2 - Percentage of proteins in COG groups for | | | | --- | --- | | Archaea | Bacteria |
- #### Supplementary figure Fig. S3 - Percentage of proteins in COG categories for | | | | --- | --- | | Archaea | Bacteria |
- #### Supplementary Figure S4 - Disorder content of different data subsets for | | | | | | --- | --- | --- | --- | | Archaea | percentage of disordered AA | percentage of disordered AA in long(>30) disordered regions | percentage of proteins with long(>30) disordered regions | Bacteria | percentage of disordered AA | percentage of disordered AA in long(>30) disordered regions | percentage of proteins with long(>30) disordered regions |
- #### Supplementary Figure S5 - Disorder content in long (>30AA) disordered regions in Archaea and Bacteria by gene location, as a function of genome size, proteome size, average protein length and G+C content. | | | | | | --- | --- | --- | --- | | Genome size | Proteome size | Average protein length | G+C content | Disorder is predicted by the IsUnstruct predictor.
- #### Supplementary Figure S6 - Percentage of disordered AA, disordered AA in long (>30AA) disordered regions and percentage of proteins containing long disordered regions for different groups of clusters of orthologous groups of proteins (COG groups) in Archaea and Bacteria | | | | | --- | --- | --- | | Percentage od disordered AA | Percentage of AA in long (>30AA) disordered regions | Percentage of proteins in long (>30AA) disordered regions | Disorder is predicted by the IsUnstruct predictor.
- #### Supplementary Figure S7 - Protein percentage in COG groups over group of material
- #### Supplementary Figure S8 - Disorder content of different COG groups and data subsets for | | | | | | --- | --- | --- | --- | | Archaea | percentage of disordered AA | percentage of disordered AA in long(>30) disordered regions | percentage of proteins with long(>30) disordered regions | Bacteria | percentage of disordered AA | percentage of disordered AA in long(>30) disordered regions | percentage of proteins with long(>30) disordered regions |
- #### Supplementary Figure S9 - Disorder content of different COG categories and data subsets for | | | | | | --- | --- | --- | --- | | Archaea | percentage of disordered AA | percentage of disordered AA in long(>30) disordered regions | percentage of proteins with long(>30) disordered regions | Bacteria | percentage of disordered AA | percentage of disordered AA in long(>30) disordered regions | percentage of proteins with long(>30) disordered regions |

  Some comments for Bacteria and percentage of disordered AA in long disordered regions:

  **(a)**
  Within the Cellular processing and signaling (Cp) COG group, the three most IDP-abundant categories for both chromosome- and plasmid-encoded proteins are Cell motility (N), Cell cycle control, cell division, chromosome partitioning (D) and Intracellular trafficking, secretion, and vesicular transport (U) COG categories. COG categories Extracellular structures (W), Cytoskeleton (Z) and Nuclear structure (Y) cannot be considered due to small number of representative proteins in each category. In all mentioned categories plasmid encoded proteins have equal or smaller IDP content than chromosomal proteins.

  **(b)**
  Within the Information storage and processing (Isp) group, the most IDP-abundant categories are Translation, ribosomal structure and biogenesis (J), Replication, recombination and repair (L) and Transcription (K) categories. Plasmid encoded proteins have significantly lower IDP content in J category, while in K and L categories it is slightly higher. RNA processing and modification (A) and Chromatin structure and dynamics (B) categories, cannot be considered due to small number of representative proteins in each category.

  **(c)**
  Within Metabolism (Me) group, all categories possess lower disorder content than in Cp and Isp groups. Disorder content is almost the same for plasmid- and chromosome-encoded proteins, for most of the categories (for Energy production and conversion COG (C), Amino acid transport and metabolism (E), Carbohydrate transport and metabolism (G), Lipid transport and metabolism (I), Inorganic ion transport and metabolism (P) and Secondary metabolites biosynthesis, transport, and catabolism (Q)) except for categories Nucleotide transport and metabolism (F) and Coenzyme transport and metabolism (H), where it is higher in plasmids.

  **(d)**
  Groups Not in COGs (N.C.) and Poorly characterized (Pc) are abundant in IDP content (much more than Me and comaprable to Cp and Isp). The percentage of plasmid-encoded proteins in N.C. group is notably high - 165,579/240,245 = 69% (56% for chromosomal proteins), and IDP content of plasmidial proteins is higher than in chromosomal proteins.
- #### Supplementary Figure S10 - Disorder content in hypothetical proteins in comparison to non-hypothetical proteins | | | | --- | --- | | Archaea | Bacteria |
- #### Supplementary Figure S11 - Disorder content in Archaea and Bacteria (data subsets for organisms with at most 20% of their proteins belonging to the N.C. group): three predictors with three measures
- #### Supplementary Figure S12 - Disorder content of different data subsets for organisms where genomes with at most 20% of their proteins belonging to the N.C. group for | | | | | | --- | --- | --- | --- | | Archaea | percentage of disordered AA | percentage of disordered AA in long(>30) disordered regions | percentage of proteins with long(>30) disordered regions | Bacteria | percentage of disordered AA | percentage of disordered AA in long(>30) disordered regions | percentage of proteins with long(>30) disordered regions |
- #### Supplementary figure Fig. S13 - Disorder content of different COG groups and data subsets for organisms where genomes with at most 20% of their proteins belonging to the N.C. group for | | | | --- | --- | | Archaea | Bacteria |
- #### Supplementary Figure S14 - Average disorder level and protein length of Toxin/Antitoxin proteins in chromosomes and plasmids
- #### Supplementary Figure S15 - Average protein length and disorder level of Toxin and Antitoxin proteins over COG groups in complete genomes. Predictor: IsUnstruct, Measure: length 1.
- #### Supplementary figure Fig. S16 - Average percentage of disorder, protein length of toxin/antitoxin/non-toxin proteins in in COG groups over chromosomes and plasmids for | | | | --- | --- | | Archaea | Bacteria |

---

## ---

 
