# Supplementary figures and images for: Structural disorder of plasmid-encoded proteins in Bacteria and Archaea
Source: BMC Bioinformatics. 2018 Apr 25;19:158. doi: 10.1186/s12859-018-2158-6 (PMC5922023; doi:10.1186/s12859-018-2158-6)

## Percentage of proteins in COG groups for Archaea

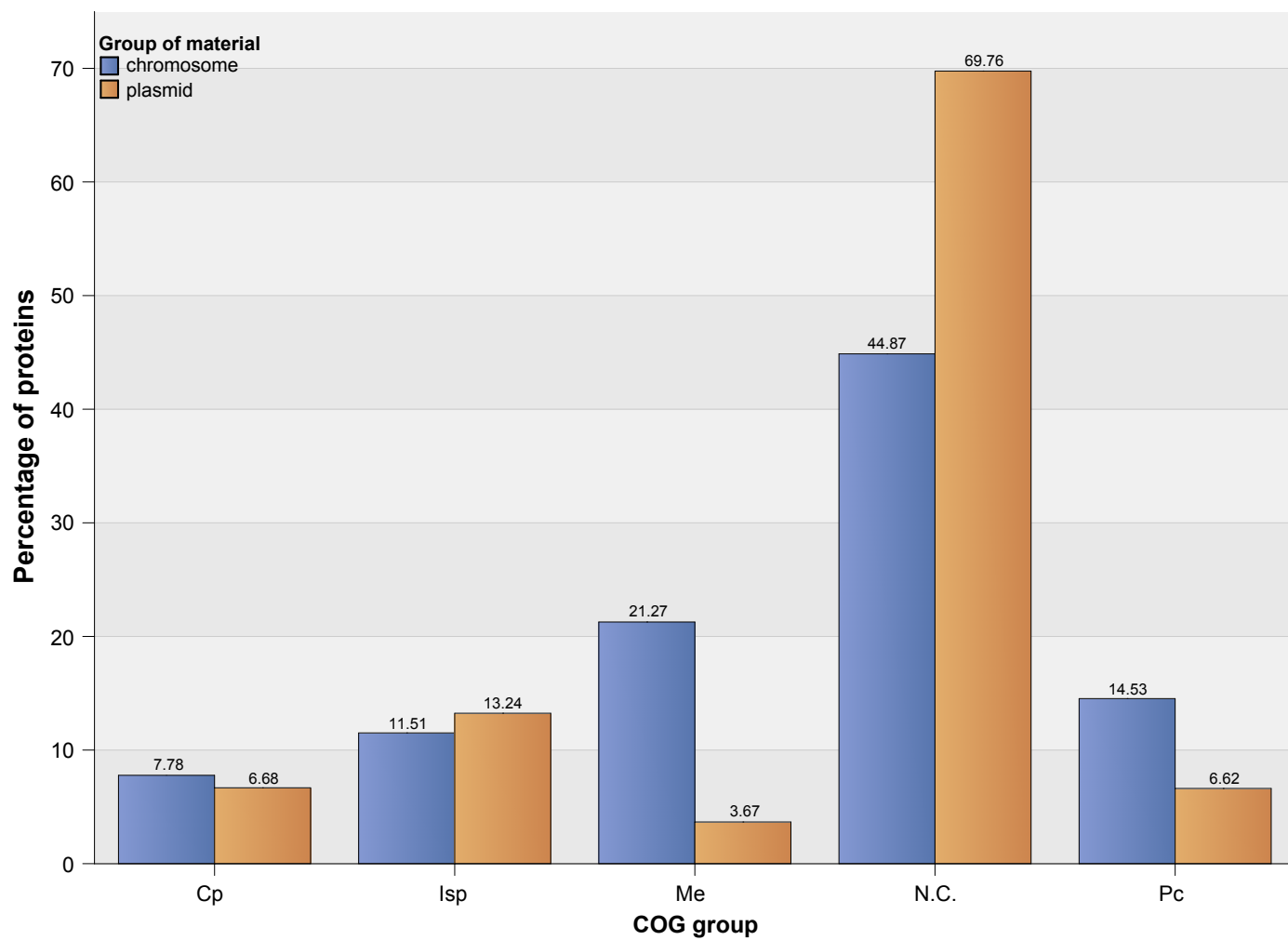

Supplement: Supplementary file 1 — This file includes additional tables and figures not shown in the manuscript. (ZIP 6200 kb) [file 12859_2018_2158_MOESM1_ESM.zip › Supplementary/s.figure2/s.figure_2._archaea_cog_percent_prot.pdf]

## Percentage of proteins in COG groups for Bacteria

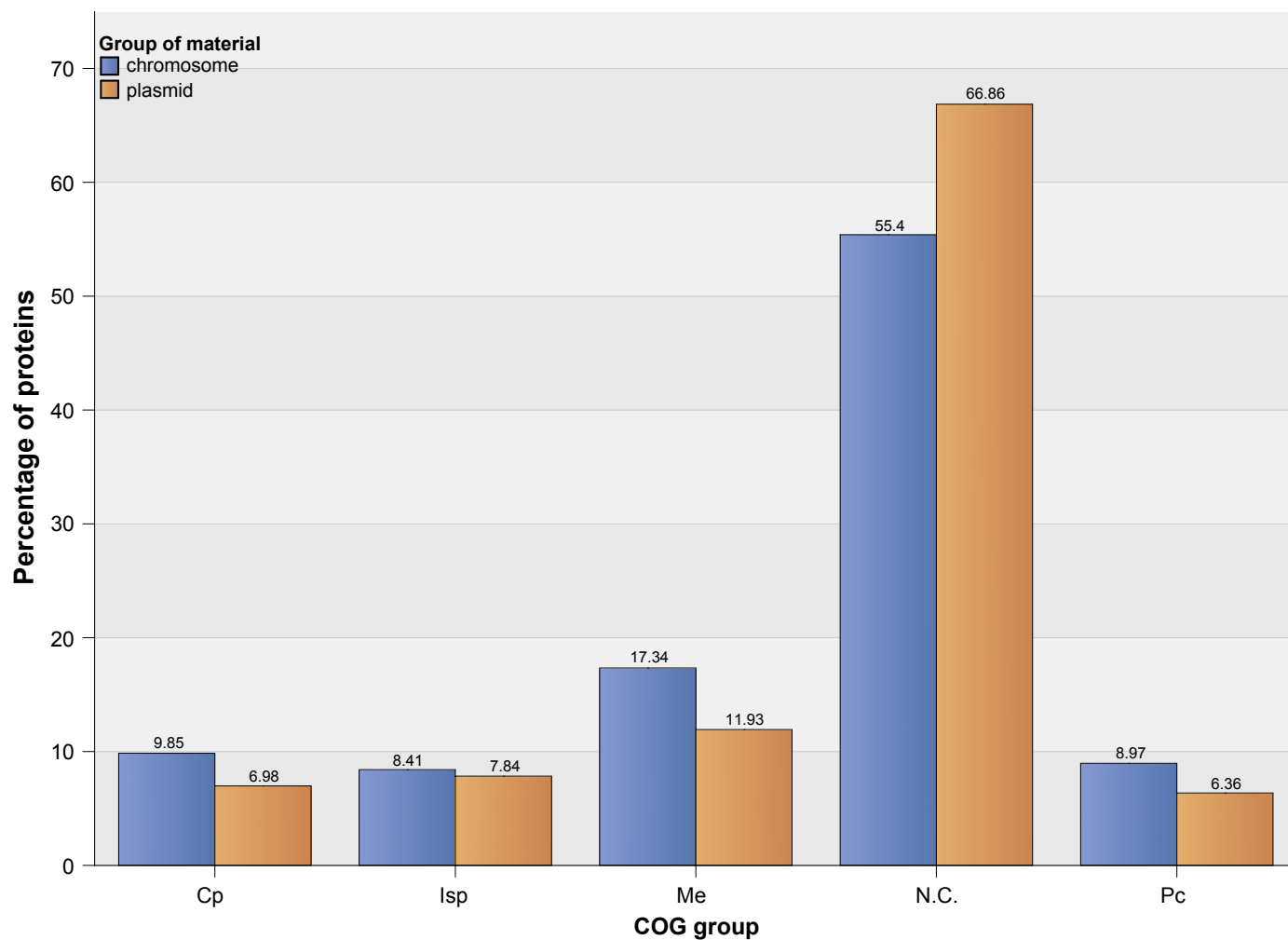

Supplement: Supplementary file 1 — This file includes additional tables and figures not shown in the manuscript. (ZIP 6200 kb) [file 12859_2018_2158_MOESM1_ESM.zip › Supplementary/s.figure2/s.figure_2._bacteria_cog_percent_prot.pdf]

## Percentage of disorder content in proteins in different data subsets for Archaea

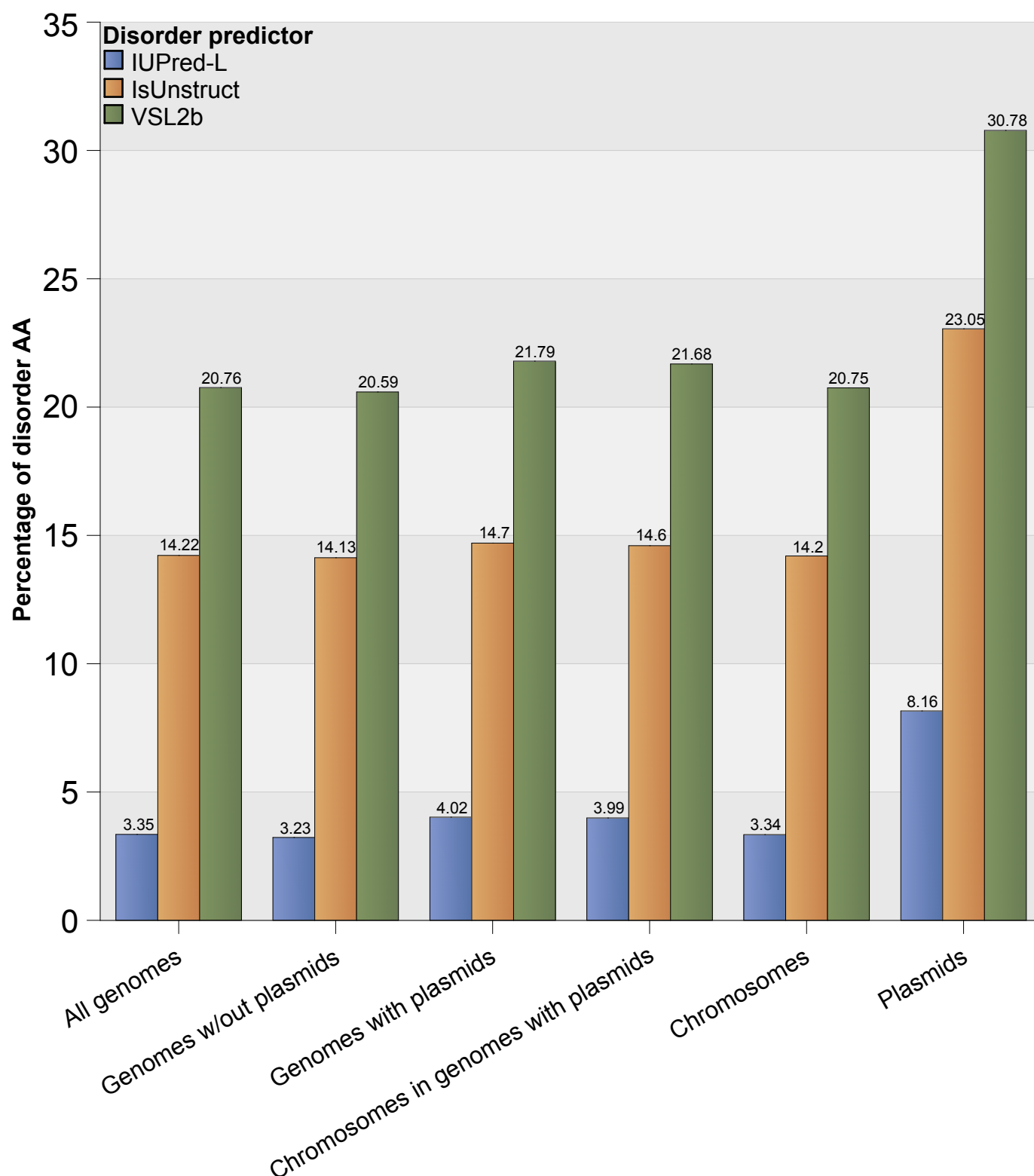

Supplement: Supplementary file 1 — This file includes additional tables and figures not shown in the manuscript. (ZIP 6200 kb) [file 12859_2018_2158_MOESM1_ESM.zip › Supplementary/s.figure4/s.figure_4._archaea_dis_1.pdf]

## Disorder content in long(>30) IDR in proteins in different data subsets in Archaea

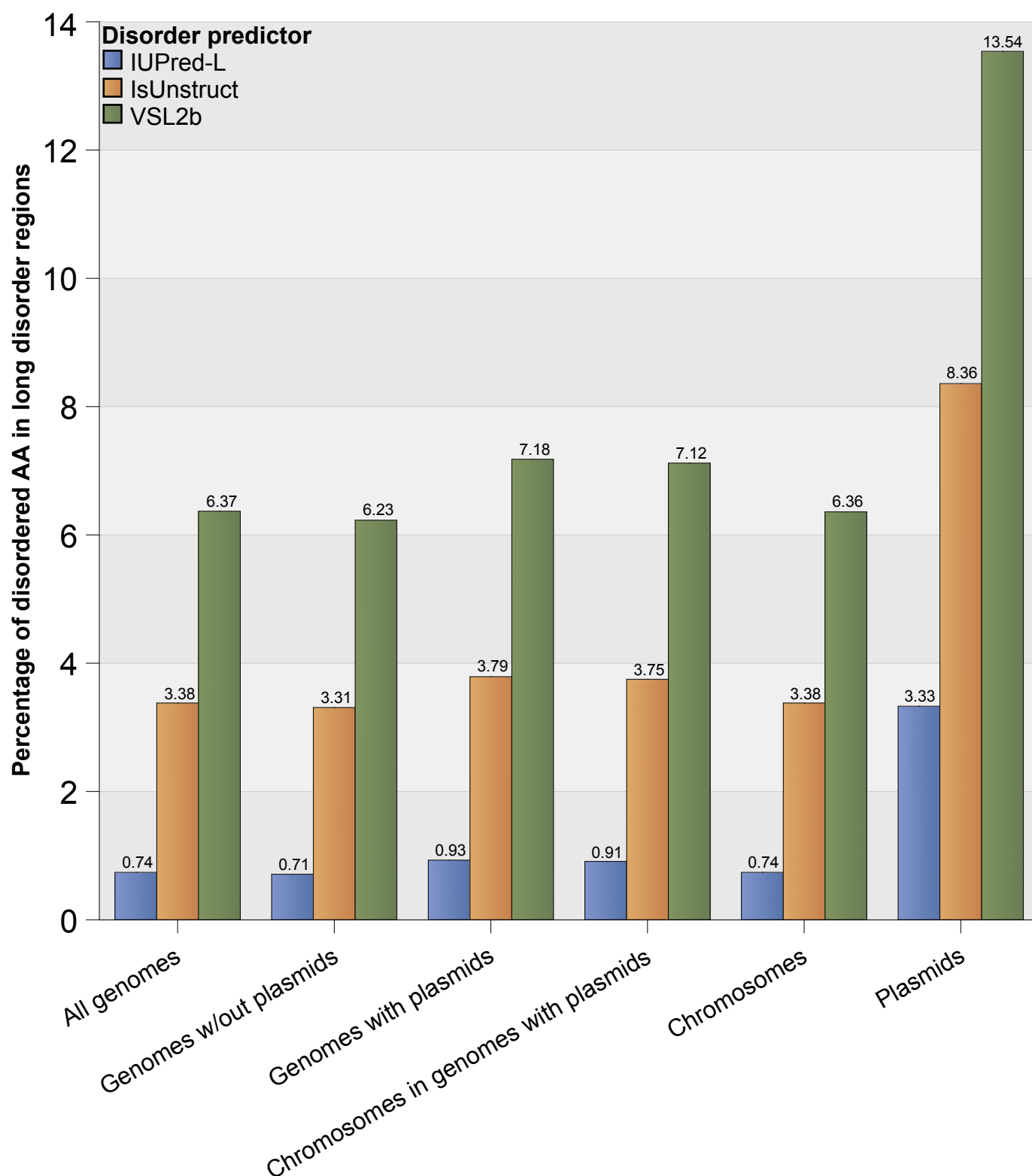

Supplement: Supplementary file 1 — This file includes additional tables and figures not shown in the manuscript. (ZIP 6200 kb) [file 12859_2018_2158_MOESM1_ESM.zip › Supplementary/s.figure4/s.figure_4._archaea_dis_31.pdf]

## Percentage of proteins with long(>30) IDRs in different data subsets in Archaea

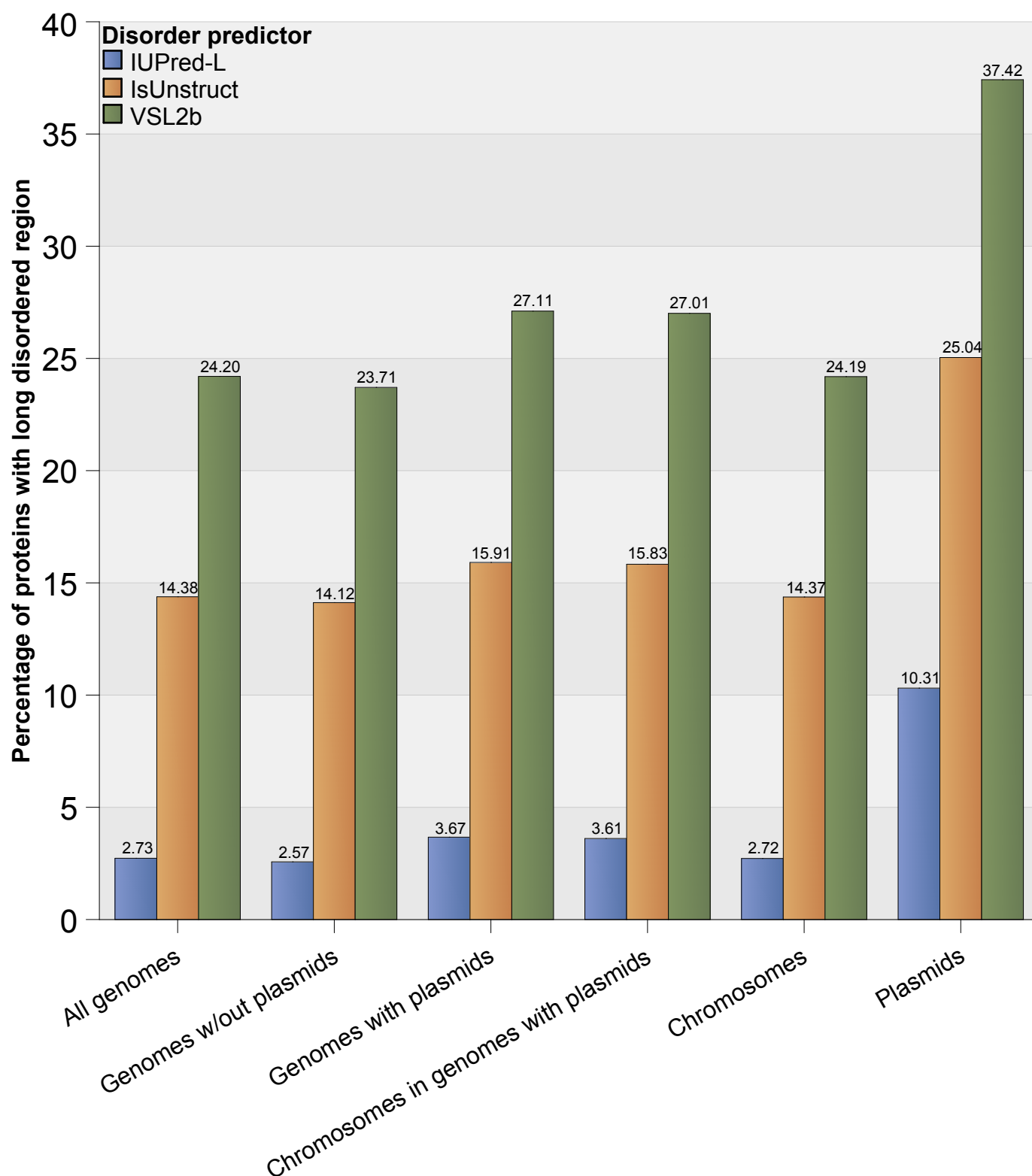

Supplement: Supplementary file 1 — This file includes additional tables and figures not shown in the manuscript. (ZIP 6200 kb) [file 12859_2018_2158_MOESM1_ESM.zip › Supplementary/s.figure4/s.figure_4._archaea_perc_prot_dis_31.pdf]

## Percentage of disorder content in proteins in different data subsets for Bacteria

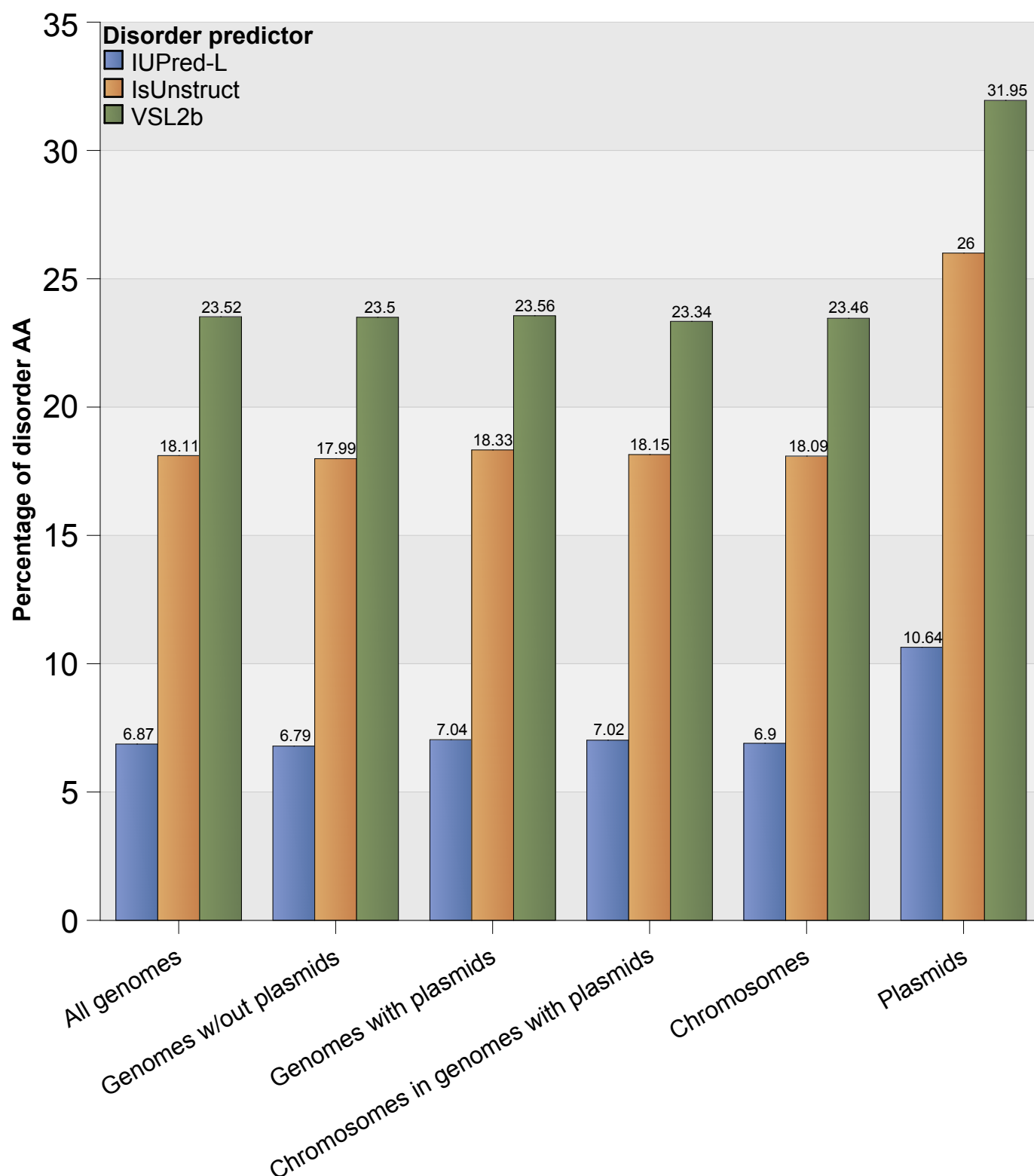

Supplement: Supplementary file 1 — This file includes additional tables and figures not shown in the manuscript. (ZIP 6200 kb) [file 12859_2018_2158_MOESM1_ESM.zip › Supplementary/s.figure4/s.figure_4._bacteria_dis_1.pdf]

## Disorder content in long(>30) IDR in proteins in different data subsets in Bacteria

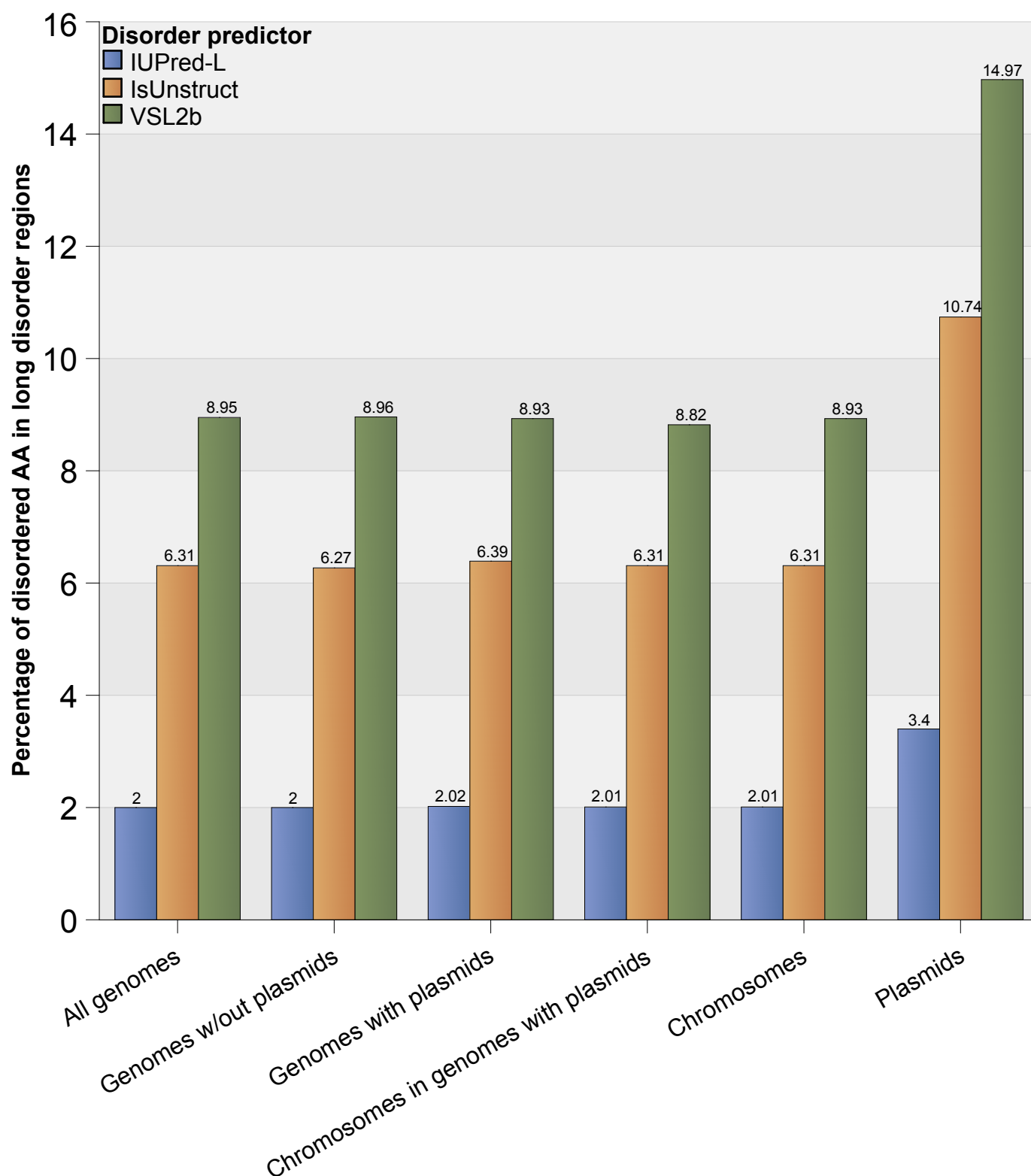

Supplement: Supplementary file 1 — This file includes additional tables and figures not shown in the manuscript. (ZIP 6200 kb) [file 12859_2018_2158_MOESM1_ESM.zip › Supplementary/s.figure4/s.figure_4._bacteria_dis_31.pdf]

## Percentage of proteins with long(>30) IDRs in different data subsets in Bacteria

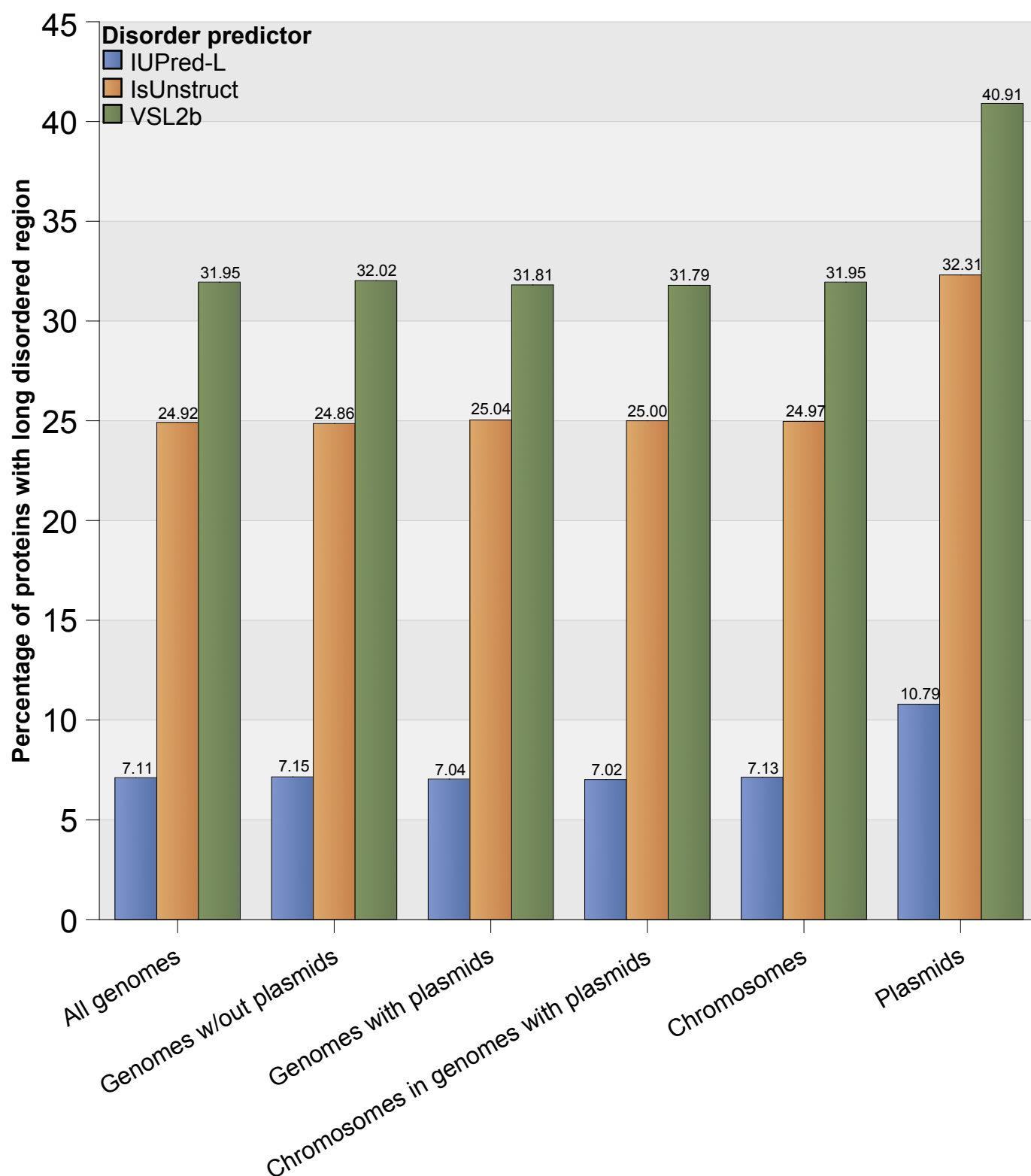

Supplement: Supplementary file 1 — This file includes additional tables and figures not shown in the manuscript. (ZIP 6200 kb) [file 12859_2018_2158_MOESM1_ESM.zip › Supplementary/s.figure4/s.figure_4._bacteria_perc_prot_dis_31.pdf]

# Disorder content of different COG groups and data subsets for Archaea

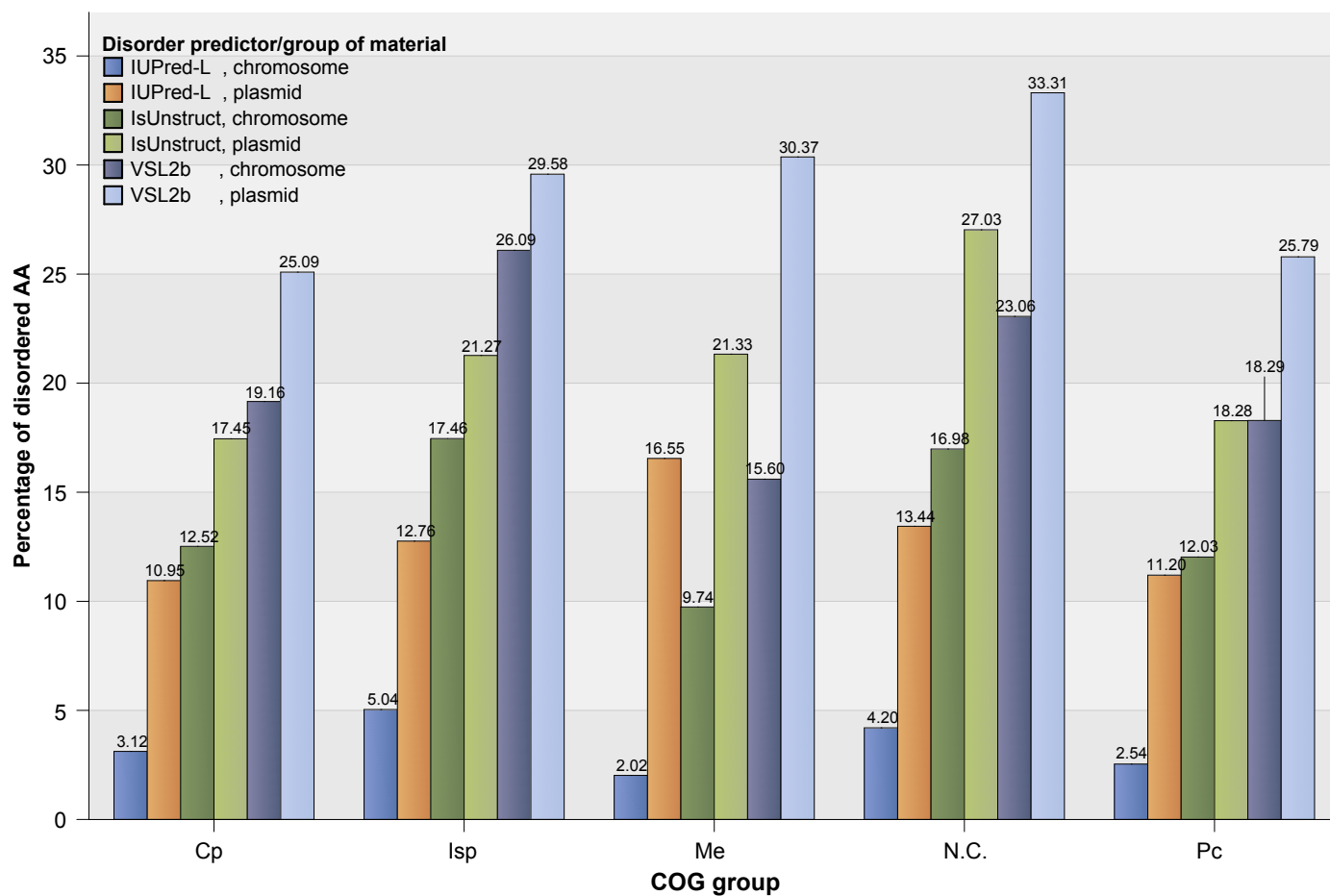

Supplement: Supplementary file 1 — This file includes additional tables and figures not shown in the manuscript. (ZIP 6200 kb) [file 12859_2018_2158_MOESM1_ESM.zip › Supplementary/s.figure8/s.figure_8._archaea_dis_1.pdf]

# Disorder content of different COG groups and data subsets for Bacteria

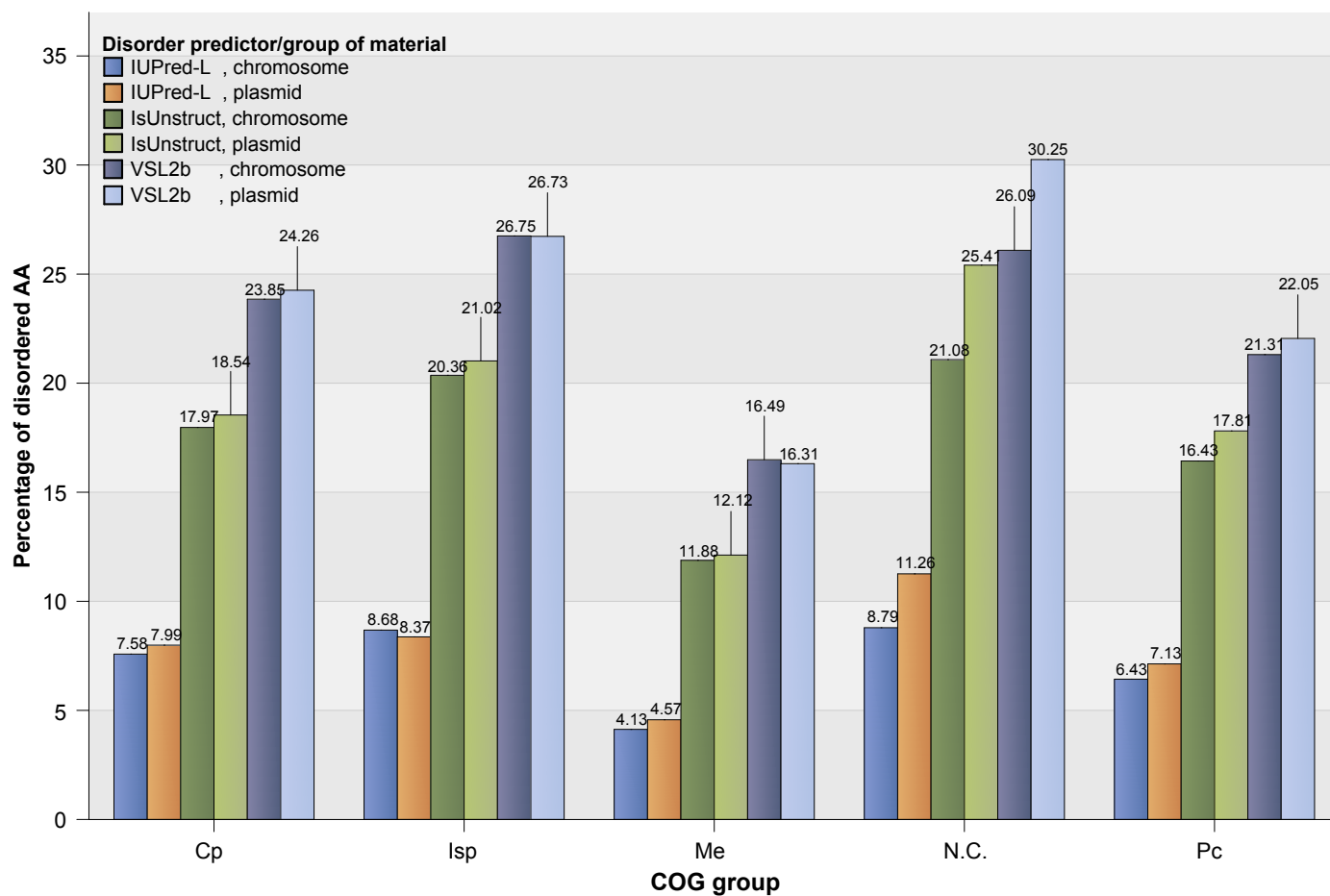

Supplement: Supplementary file 1 — This file includes additional tables and figures not shown in the manuscript. (ZIP 6200 kb) [file 12859_2018_2158_MOESM1_ESM.zip › Supplementary/s.figure8/s.figure_8._bacteria_dis_1.pdf]

### Percentage of proteins in COG category with long(>30AA) disordered regions in Bacteria

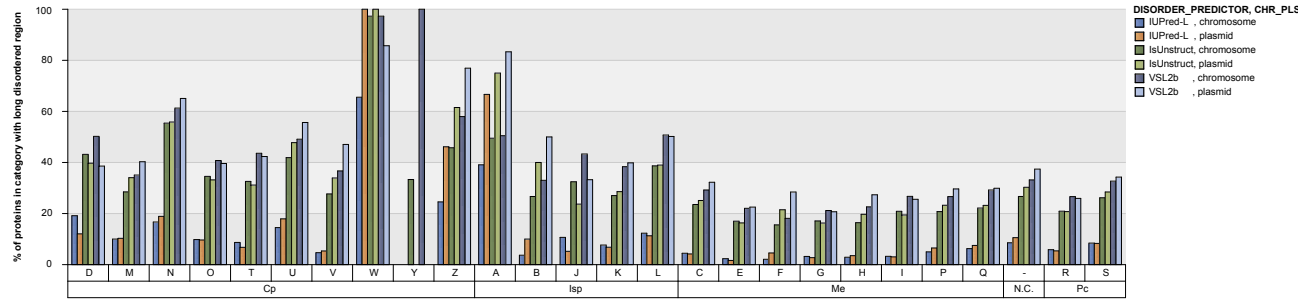

Supplement: Supplementary file 1 — This file includes additional tables and figures not shown in the manuscript. (ZIP 6200 kb) [file 12859_2018_2158_MOESM1_ESM.zip › Supplementary/s.figure9/s.figure_9._bacteria_perc_prot_dis_31.pdf]
